# The Role of *Abcb5* Alleles in Susceptibility to Haloperidol-Induced Toxicity in Mice and Humans

**DOI:** 10.1371/journal.pmed.1001782

**Published:** 2015-02-03

**Authors:** Ming Zheng, Haili Zhang, David L. Dill, J. David Clark, Susan Tu, Arielle L. Yablonovitch, Meng How Tan, Rui Zhang, Dan Rujescu, Manhong Wu, Lino Tessarollo, Wilfred Vieira, Michael M. Gottesman, Suhua Deng, Livia S. Eberlin, Richard N. Zare, Jean-Martin Billard, Jean-Pierre Gillet, Jin Billy Li, Gary Peltz

**Affiliations:** 1 Department of Anesthesia, Stanford University School of Medicine, Stanford, California, United States of America; 2 Computer Science, Stanford University, Stanford, California, United States of America; 3 Veterans Affairs Palo Alto Health Care System, Palo Alto, California, United States of America; 4 Department of Genetics, Stanford University School of Medicine, Stanford, California, United States of America; 5 Department of Psychiatry, University Of Halle, Halle, Germany; 6 Center for Cancer Research, National Cancer Institute, Frederick, Maryland, United States of America; 7 Laboratory of Cell Biology, National Cancer Institute, National Institutes of Health, Bethesda, Maryland, United States of America; 8 Department of Pathology, Stanford University, Stanford, California, United States of America; 9 Department of Chemistry, Stanford University, Stanford, California, United States of America; 10 Laboratory of Molecular Cancer Biology, Molecular Physiology Research Unit (URPhyM), Namur Research Institute for Life Sciences (NARILIS), Faculty of Medicine, University of Namur, Belgium; University of Liverpool, UNITED KINGDOM

## Abstract

**Background:**

We know very little about the genetic factors affecting susceptibility to drug-induced central nervous system (CNS) toxicities, and this has limited our ability to optimally utilize existing drugs or to develop new drugs for CNS disorders. For example, haloperidol is a potent dopamine antagonist that is used to treat psychotic disorders, but 50% of treated patients develop characteristic extrapyramidal symptoms caused by haloperidol-induced toxicity (HIT), which limits its clinical utility. We do not have any information about the genetic factors affecting this drug-induced toxicity. HIT in humans is directly mirrored in a murine genetic model, where inbred mouse strains are differentially susceptible to HIT. Therefore, we genetically analyzed this murine model and performed a translational human genetic association study.

**Methods and Findings:**

A whole genome SNP database and computational genetic mapping were used to analyze the murine genetic model of HIT. Guided by the mouse genetic analysis, we demonstrate that genetic variation within an ABC-drug efflux transporter *(Abcb5)* affected susceptibility to HIT. In situ hybridization results reveal that Abcb5 is expressed in brain capillaries, and by cerebellar Purkinje cells. We also analyzed chromosome substitution strains, imaged haloperidol abundance in brain tissue sections and directly measured haloperidol (and its metabolite) levels in brain, and characterized *Abcb5* knockout mice. Our results demonstrate that *Abcb5* is part of the blood-brain barrier; it affects susceptibility to HIT by altering the brain concentration of haloperidol. Moreover, a genetic association study in a haloperidol-treated human cohort indicates that human *ABCB5* alleles had a time-dependent effect on susceptibility to individual and combined measures of HIT. *Abcb5* alleles are pharmacogenetic factors that affect susceptibility to HIT, but it is likely that additional pharmacogenetic susceptibility factors will be discovered.

**Conclusions:**

*ABCB5* alleles alter susceptibility to HIT in mouse and humans. This discovery leads to a new model that (at least in part) explains inter-individual differences in susceptibility to a drug-induced CNS toxicity.

## Abstract 2

Gary Peltz and colleagues examine the role of ABCB5 alleles in haloperidol-induced toxicity in a murine genetic model and humans treated with haloperidol.

## Editors' Summary

**Background:**

The brain is the control center of the human body. This complex organ controls thoughts, memory, speech, and movement, it is the seat of intelligence, and it regulates the function of many organs. The brain comprises many different parts, all of which work together but all of which have their own special functions. For example, the forebrain is involved in intellectual activities such as thinking whereas the hindbrain controls the body’s vital functions and movements. Messages are passed between the various regions of the brain and to other parts of the body by specialized cells called neurons, which release and receive signal molecules known as neurotransmitters. Like all the organs in the body, blood vessels supply the brain with the oxygen, water, and nutrients it needs to function. Importantly, however, the brain is protected from infectious agents and other potentially dangerous substances circulating in the blood by the “blood-brain barrier,” a highly selective permeability barrier that is formed by the cells lining the fine blood vessels (capillaries) within the brain.

**Why Was This Study Done?:**

Although drugs have been developed to treat various brain disorders, more active and less toxic drugs are needed to improve the treatment of many if not most of these conditions. Unfortunately, relatively little is known about how the blood-brain barrier regulates the entry of drugs into the brain or about the genetic factors that affect the brain’s susceptibility to drug-induced toxicities. It is not known, for example, why about half of patients given haloperidol—a drug used to treat psychotic disorders (conditions that affect how people think, feel, or behave)—develop tremors and other symptoms caused by alterations in the brain region that controls voluntary movements. Here, to improve our understanding of how drugs enter the brain and impact its function, the researchers investigate the genetic factors that affect haloperidol-induced toxicity by genetically analyzing several inbred mouse strains (every individual in an inbred mouse strain is genetically identical) with different susceptibilities to haloperidol-induced toxicity and by undertaking a human genetic association study (a study that looks for non-chance associations between specific traits and genetic variants).

**What Did the Researchers Do and Find?:**

The researchers used a database of genetic variants called single nucleotide polymorphisms (SNPs) and a computational genetic mapping approach to show first that variations within the gene encoding Abcb5 affected susceptibility to haloperidol-induced toxicity (indicated by changes in the length of time taken by mice to move their paws when placed on an inclined wire-mesh screen) among inbred mouse strains. Abcb5 is an ATP-binding cassette transporter, a type of protein that moves molecules across cell membranes. The researchers next showed that Abcb5 is expressed in brain capillaries, which is the location of the blood-brain barrier. Abcb5 was also expressed in cerebellar Purkinje cells, which help to control motor (intentional) movements. They also measured the measured the effect of haloperidol and the haloperidol concentration in brain tissue sections in mice that were genetically engineered to make no Abcb5 (*Abcb5* knockout mice). Finally, the researchers investigated whether specific alleles (alternative versions) of *ABCB5* are associated with haloperidol-induced toxicity in people. Among a group of 85 patients treated with haloperidol for a psychotic illness, one specific *ABCB5* allele was associated with haloperidol-induced toxicity during the first few days of treatment.

**What Do These Findings Mean?:**

These findings indicate that Abcb5 is a component of the blood-brain barrier in mice and suggest that genetic variants in the gene encoding this protein underlie, at least in part, the differences in susceptibility to haloperidol-induced toxicity seen among inbred mice strains. Moreover, the human genetic association study indicates that a specific *ABCB5* allele also affects the susceptibility of people to haloperidol-induced toxicity. The researchers note that other *ABCB5* alleles or other genetic factors that affect haloperidol-induced toxicity in people might emerge if larger groups of patients were studied. However, based on their findings, the researchers propose a new model for the genetic mechanisms that underlie inter-individual and cell type-specific differences in susceptibility to haloperidol-induced brain toxicity. If confirmed in future studies, this model might facilitate the development of more effective and less toxic drugs to treat a range of brain disorders.

**Additional Information:**

Please access these websites via the online version of this summary at http://dx.doi.org/10.1371/journal.pmed.1001782.

- The US National Institute of Neurological Disorders and Stroke provides information about a wide range of brain diseases (in English and Spanish); its fact sheet “Brain Basics: Know Your Brain” is a simple introduction to the human brain; its “Blueprint Neurotherapeutics Network” was established to develop new drugs for disorders affecting the brain and other parts of the nervous system
- MedlinePlus provides links to additional resources about brain diseases and their treatment (in English and Spanish)
- Wikipedia provides information about haloperidol, about ATP-binding cassette transporters and about genetic association (note that Wikipedia is a free online encyclopedia that anyone can edit; available in several languages)

## Introduction

Relatively little is known about the mechanisms regulating the entry of many drugs into the brain, or about the genetic mechanisms affecting susceptibility to many drug-induced central nervous system (CNS) toxicities. Because of this lack of information, candidate drugs affecting the CNS have the lowest rate of survival during clinical development (<2%), and drug distribution across the blood-brain barrier has been identified as a contributing cause for drug candidate failure [1]. The blood-brain barrier is a very complex multicellular vascular structure, which separates the brain from the peripheral blood by actively controlling the entry and efflux of drugs [2]. Therefore, the mechanistic insight obtained by identification of genetic factors affecting a drug-induced CNS toxicity could aid in the development of new medicines and could improve our ability to optimally utilize existing treatments. For example, haloperidol is a potent dopamine receptor antagonist that is used to treat psychotic disorders [3]. However, haloperidol-induced alterations in the extrapyramidal motor system depress the ability to initiate voluntary movements. This effect triggers the characteristic extrapyramidal symptoms (EPS) that develop in 40%–76% of chronically treated human patients, which include tremors, Parkinsonian rigidity, and decreased spontaneous movement [4,5]. The same symptoms appear in some (but not all) of the 27 inbred strains that were treated with haloperidol [6]. In this murine genetic model, the time required (latency) for a mouse to move all four paws after being placed on an inclined wire-mesh screen was measured as an index of the haloperidol-induced Parkinsonian rigidity that is observed in haloperidol-treated human patients. Although all strains had plasma drug levels that were similar to those measured in human patients, the extent of the haloperidol-induced toxicity (HIT) varied in a highly (85%) heritable manner [6] among the inbred strains.

Because no pharmacogenetic factors for HIT have previously been identified in humans or mice, we wanted to analyze this murine genetic model of HIT. Of concern, mouse genetic studies have, overall, produced rather disappointing results; they have definitively identified <1% of the genes affecting the studied traits [7]. Beyond the well described problems associated with murine linkage studies [8], several analyses have predicted that the conventional methods used to analyze genome wide association studies (GWAS) using inbred mouse strains will produce even more disappointing results, owing to their low power and a high false positive rate [7,9,10]. However, the availability of next generation sequence (NGS) data, which can provide complete genome-wide variant information for an expanded number of inbred strains [11], could enable murine GWAS to identify causative genetic factors for many biomedical traits [12]. The detailed genetic variation information obtained using NGS has increased power for pinpointing underlying causal variants relative to studies using SNP array data [13]. Unlike the conventional murine GWAS methods, haplotype-based computational genetic mapping (HBCGM) [14] has provided an alternative method for analysis of murine GWAS data that has identified causative genetic factors for many biomedical traits (reviewed in [12]). HBCGM first organizes the allelic information into haplotype blocks, which facilitates the identification of the causative genetic factors through correlation of the phenotypic data with the haplotype-based pattern of genetic variation. Since our prior studies analyzed much less complex datasets, which had fewer SNPs and haplotype blocks, we did not know if HBCGM could analyze the far more complex datasets produced using NGS data. To determine if HBCGM could be successfully combined with an SNP database generated from whole genome sequence data, we analyzed a murine genetic model of HIT in order to identify causative genetic factors. A translational human genetic association study was then performed to determine if similar genetic factors affected human susceptibility to HIT.

## Methods

### Next-Generation Sequencing of the Genome of Nine Inbred Mouse Strains

Genomic DNA was obtained from the Jackson Laboratory (http://www.jax.org). A total of 3 μg of DNA from each of nine inbred mouse strains was sonicated using a Covaris instrument with the following settings: duty cycle, 10%; intensity, 4; cycles per burst, 200; and time, 60 seconds. After end repair of the DNA fragments and addition of “A” base to the 3′ ends, standard Illumina genomic DNA sequencing adaptors were ligated. The samples were run on a 3% agarose gel, gel slices between 300–500 bp were excised, and the DNA was purified with a Qiagen column. The DNA was divided into three aliquots of equal quantity, and amplified separately using one of the following three DNA polymerases: Phusion HF (Finnzymes), Kapa HiFi (Kapa Biosystems), and Herculase (Agilent). For analysis of the SJL strain, Phusion GC (Finnzymes) was also used to amplify a separate aliquot. The PCR conditions for amplification were as follows: 98°C for 3 minutes, followed by nine cycles of 98°C for 20 seconds, 62°C for 30 seconds, and 72°C for 45 seconds, and then a final 72°C for 5 minutes. The amplified products were separated on an agarose gel, slices between 400 and 600 bp were excised, and the DNA was eluted from the gel slices and sequenced on an Illumina HiSeq 2000 instrument.

### Sequence Analysis and Variant Calling

The quality statistics for the allele calls at each position were evaluated using the FASTX-Toolkit (http://hannonlab.cshl.edu/fastx_toolkit/). A rapid decay in sequence quality was observed toward the end of each read, usually within the last 10 (or sometimes 20) bps. To avoid artifacts caused by low quality sequence data, the last 10 or 20 bps of each read were trimmed, based upon a visual inspection of the quality statistics plot for the reads in each lane. The extent of trimming was designed to ensure that the quality scores should be ≥30 in the 1st quartile and ≥20 in the 5th percentile. All sequences were trimmed by 10 bps; with the exceptions being one of the three lanes for the MA/MyJ and MRL, and two of the three SM/J lanes. Since a much larger number of sequences were obtained for SJL, SJL sequences were trimmed by 20 bps to ensure the overall quality of the base calls. The read sequences were then aligned to the reference C57BL/6J mouse genome sequence (MGSCv37 assembly) using BWA [15]. Optical and PCR duplicates were then marked using the Picard tool (http://picard.sourceforge.net/). The sequence variants (SNPs and INDELs) for each strain were identified using Samtools [16], and the default settings were applied. The raw sequence data for the 17 inbred mouse strains described in [11] were obtained from the European Nucleotide Archive (http://www.ebi.ac.uk/ena/), and the same data processing procedures were applied, but these sequences were not trimmed.

To ensure high quality variant calls, the variants were further filtered using two additional criteria. (i) To ensure a high level of confidence in the existence of the variant, we require the QUAL score, which represents the (Phred-scaled) probability that all observed samples are homozygous reference alleles, should be ≥50. (ii) To ensure a high level of confidence that the variant allele is indeed homozygous, the genotype call (the “GT” value) must be a homozygous alternative call, and that the Phred-scaled genotype likelihood (the “PL” value) for the homozygous alternative call is at least 20 units above that of any other PL value. If the variant allele call for a strain does not meet these criteria, the call is converted to an “N” (undetermined) instead of being disregarded, which ensures that we don’t mistakenly assume that it is the reference allele. Information about the fold-coverage and other aspects of the sequencing methods are provided in S1 Text.

### Haplotype Block Construction and Genetic Mapping in Mice

HBCGM utilizes bi-allelic SNPs that are polymorphic among the strains. Only bi-allelic SNPs with at least one definitive homozygous alternative call among the 23 strains were included, while all other variants (including INDELs) were marked and excluded from HBCGM analysis. The locations and potential codon-changes caused by the SNPs are then annotated relative to the encoded genes using predictive gene models from Ensembl version 65. Only SNPs meeting the following criteria were used for haplotype block construction: (i) polymorphic among the strains with input trait data; and (ii) there were at least eight strains (or one-half of the number of input strains) with unambiguous allele calls. Haplotype blocks with 2, 3, 4, or haplotypes were then dynamically produced as described in [17], and the correlation between the input phenotypic data and the haplotype pattern within each identified block was evaluated as described [14]. The genes are then sorted based upon the ANOVA *p*-value (in increasing order) for numeric data or by the F statistic (in decreasing order) for categorical data. For a gene that is covered by multiple blocks, the smallest *p*-value (or the largest F statistic) obtained for all blocks within that gene was used.

The genetic effect size is calculated using our previously described method [18]: Genetic effect size = (SSB − (*k* − 1) * MSE)/(SStotal + MSE), where SSB is the between-group sum-of-squares of the ANOVA model, SStotal is the total sum-of-squares, *k* is the number of groups, and MSE is within-group sum-of-squares divided by (*n* − *k*), where *n* is the total number of objects in the model. Given the very large number of haplotype blocks produced by the HBCGM method, all results were automatically filtered according to pre-determined criteria using software that selected the correlated genes that were expressed in a designated target organ and had missense SNPs.

### Robustness Assessment

To assess the robustness of the HBCGM output obtained using the data obtained from different sets of input strains, we developed software that would automatically perform a re-sampling analysis. For each iteration, a number (1, 2, or 3) was randomly selected to indicate the number of strains whose data would be randomly excluded from the 17 strains with available latency data. Next, the selected number of strains (and each of the individual strains to be deleted were also randomly selected) was excluded for this iteration. Then, HBCGM were run on this re-sampled data and the result was recorded (using *p* = 0.01 as the cutoff). This process was repeated 100 times. If a re-sampled strain panel was identical to one that had already been re-sampled, this iteration was disregarded, and another subset was randomly chosen using the same procedure. To measure the overall association of a gene with the phenotype among the 100 random re-sampled sets of output, we defined a robustness score based on the *p*-values obtained, which was calculated as: $\text{Score}=\sum_{i=1}^{100}{\text{-log}}_{10}(p_{i})$, where *p*_*i*_ was the resulting *p*-value for that gene in the ith re-sampled experiment (if the *p*-value was bigger than the cutoff and therefore censored in the result, *p*_*i*_ was set to 0.01, i.e., the cutoff). Genes with a larger robustness index have stronger overall correlation among the different subsets analyzed. Their calculated scores then were used to rank the genes, and those with the highest scores were output.

### Statistical Analysis of Haloperidol Latency Data in Mice

The haloperidol-induced latency data measured on days 0, 3, 7, 30, 60, and 120 (MPD datasets: 39407, 39408, 39409, 39410, 39411, and 39445, respectively) and the plasma haloperidol concentrations measured on days 30, 60, 90, and 120 (MPD numbers: 39403–39406, respectively) were obtained from the Mouse PHENOME Database (http://phenome.jax.org/). The correlation analyses were performed using the phenotypic data for all 27 strains that were evaluated. The non-parametric Spearman’s correlation coefficient (*rho*) was calculated [19], and the statistical significance of the correlation between the tested datasets was evaluated using this result. This non-parametric test was used to avoid producing spurious results that could be caused by the non-normal distribution of the data and by the presence of extreme outlying values. The *p*-values were then adjusted for multiple testing using the Benjamin-Hochberg method [20]. To minimize the effect produced by the strain with an extreme outlying value and to better visualize the relationship of the variables, the haloperidol-induced latency data from day 7 and day 30 were log-transformed (10-based). (It should be noted that the log-transformation has no effect on the Spearman’s *rho*, nor did it affect the corresponding test result.)

### Haloperidol Toxicity Measurements

All animal experiments were performed according to protocols approved by the Stanford Institutional Animal Care and Use Committee. All mice were obtained from Jackson Labs, and were used at 10–12 weeks of age, and the results are reported according to the ARRIVE guidelines (S1 Text) [21]. Haloperidol was administered to the mice by one of two methods: (i) a haloperidol pellet (Innovative Research of America) was subcutaneously implanted, which continuously released a dosage equivalent to 3 mg/kg/day, using published methods [22]; or (ii) with 10 mg/kg haloperidol (Sigma) IP qd for measurement of drug and metabolite concentrations. The haloperidol-induced latency was measured as the time required for a mouse to move all four paws after being placed on a vertical metal mesh screen as previously described [22].

### Haloperidol Quantitation

Haloperidol was purchased from Sigma-Aldrich; haloperidol-d4 was purchased from Toronto Research Chemicals Inc.; HPLC grade acetonitrile and water were from Honeywell Burdick and Jackson International Inc.; and formic acid was purchased from Pierce Thermo Scientific.

Mouse brain tissues of known weight were homogenized in methanol using a Precelly 24 (Bertin Technologies) homogenizer. 50 μl of homogenate was aliquoted, and haloperidol-d4 was added as internal standard to a final concentration of 100 ng/ml. After the mixture was incubated with three volumes of cold acetonitrile at −20°, it was centrifuged at 14,000 rpm for 10 minutes. The supernatant was dried in a speed vacuum and re-suspended in the original volume. A standard curve was prepared using brain homogenate from an untreated mouse in a linear range from 0.5 ng/ml to 250 ng/ml. Quantitative analysis of haloperidol and its metabolite (HPP+) was performed on an Agilent accurate mass QTOF 6520A coupled with an UHPLC Infinity 1290. The analytes were separated on a Phenomenex Kinetex XB-C18 column 2.1 × 100 mm. The flow rate was 0.5 ml/min with a gradient of solvent B (acetonitrile with 0.1% formic acid; solvent A is 0.1% formic acid) from 5% to 35% in 10 minutes, then 35% to 95% in 5 minutes. Positive electrospray full scan in the range of m/z 110–1,000 spectra were collected. Data analysis was done using Agilent quantitative analysis software.

### Desorption Electrospray Ionization Mass Spectrometroscopic Imaging

High mass resolution/high mass accuracy mass spectrometry tissue imaging was performed using a lab-built desorption electrospray ionization mass spectrometry imaging (DESI-MSI) source coupled to an LTQ-Orbitrap XL mass spectrometer (Thermo Scientific). DESI-MSI was performed in the positive ion mode from *m/z* 200–500, using the orbitrap as the mass analyzer at a resolving power of 60,000. The spatial resolution of the imaging experiments was of 150 μm. A histologically compatible solvent system dimethylformamide:acetonitrile (DMF:ACN) 1:1 (v/v) was used for analysis [23] at a flow rate of 0.8 μl/min. The N_2_ pressure was set to 175 psi. After DESI-MSI, the same tissue section was subjected to hematoxylin–eosin (HE) staining. The software ImgGenerator (freeware, http://www.msimaging.net/) was used for converting raw files into 2-D images. Spatially accurate ion images were assembled using BioMap software (freeware, http://www.maldi-msi.org/). The protonated form of haloperidol was detected in the tissue sections at *m/z* 376.14679 (mass error of −1.7 ppm). The isotopic distribution of the ion at *m/z* 376.14679 was also found to agree with the molecular formula of haloperidol. DESI-MS ion images of haloperidol were compared to optical images of the same tissue in HE-stained tissue sections. The total abundance of haloperidol in each pixel from a DESI-MS ion image was extracted and averaged to obtain the average abundance of haloperidol per tissue section. This procedure was performed for each tissue section analyzed for each strain of mice. Based on the average total abundance of haloperidol for each strain, the fold change was then calculated.

### RNA In Situ Hybridization

*Abcb5* and *Drd2* probes were designed to hybridize to their corresponding mRNAs. The 741 bp *Abcb5* probe was PCR amplified from RIKEN_cDNA_clone B020023O04 using the primer set: AGGCCAGAAACAGAGGATTG; and CCTGGTAGAGCATGGCTTTG. The 1,126 bp *Drd2* probe was PCR amplified from total cDNA from a C57BL/6J mouse using the primer set: CCGTGAACCCCATCATCTAT and GGTTTGGTGCATGTATGGTG. PCR reactions were performed using Phusion High-fidelity DNA polymerase (Thermo Scientific) and the products were cloned into pCR-BluntII-TOPO vector (Life Technology). Clones with correct insertions were linearized using either BamHI or NotI (New England Biolabs) for in vitro transcription. Anti-sense and sense RNA probes were then in vitro transcribed using Riboprobe System-SP6/T7 (Promega) and DIG RNA labeling mix (Roche), DNase treated, precipitated in lithium chloride (Ambion), and stored at −80°C in aliquots. Freshly dissected mouse brain was immediately bisected sagittally, and fixed in 4% paraformaldehyde in DEPC treated phosphate buffered saline at 4°C overnight. Fixed tissues were then saturated in DEPC-treated 20% sucrose at 4°C overnight, then flash frozen in OCT compound (Tissue-Tek) and stored at −80°C. The brain sections were cut at 20 μm thickness, and serial sections were collected on Superfrost Plus glass slides (Fisher Scientific). Sets of serial sections across the whole brain were then hybridized anti-sense probes, and adjacent sections were hybridized with the sense probe (as negative controls), at 63°C in a moist chamber overnight. After washing and blocking, tissue sections were then incubated with AP-conjugated anti-DIG antibody (Roche) at 4°C overnight. Tissue sections were then washed and chromogenic reactions were then performed by incubation with the AP substrate BM Purple (Roche) at room temperature. The sections were then examined using a light microscope, and a purple/blue color indicates the localization of the targeted mRNA within the tissue.

### Statistical Analysis of Data Obtained from Inbred and Chromosome Substitution Strains

The latency measurements from chromosome substitution strain (CSS) mice were log-transformed, and a two-sample *t*-test was applied to compare the latency of CSS12 to that of C57BL/6. The differences between the latency on the log-scale and the corresponding 95% confidence intervals were evaluated, and then transformed back to the original scale. Similarly, the measured haloperidol and HPP^+^ concentrations were log-transformed and a two-sample *t*-test was applied to compare the concentration in A/J to that in C57BL/6. The differences between the latency on the log-scale and the corresponding 95% confidence intervals were evaluated and then transformed back to the original scale. For comparison of the HPP^+^ levels in A/J, CSS12, and C57BL/6 strains, the concentrations were log-transformed and an F-test was applied. The differences between the latency on the log-scale and the corresponding 95% confidence intervals were evaluated using SAS (version 9.3), and were then transformed back to the original scale.

### Analysis of Abcb5 Knockout Mice

Mice with a homozygous *Abcb5* gene deletion were prepared and bred onto the C57BL/6 by nine backcrosses, and these mice did not express *Abcb5* mRNA (JPG, MMG, and colleagues, personal communication). The latency in control wild-type littermate and *Abcb5* knockout mice were analyzed after 0 to 7 days of haloperidol 10 mg/kg/day IP administration. The statistical significance of the difference in the latency measurements between wild-type and *Abcb5* knockout mice was determined using a two-sample two-sided *t*-test on log-transformed data for each day as described in the preceding paragraph. Since gender could affect the haloperidol response, we performed a two-way ANOVA to assess the effect of gender. However, the gender effect was only significant on day 4 (*p* = 0.046) and was not significant for all of the other days. Therefore, an adjustment for the gender effect was not performed.

### Human Genetic Association Analysis: Human *ABCB5* Alleles and Haloperidol Toxicity

The study was approved by the ethics committee at the University of Munich, and was carried out in accordance with the ethical standards laid down in the 1964 Declaration of Helsinki and later revisions (S2 Text). Additional description of these participants and their characteristics can be found elsewhere [24]. Written informed consent was obtained at the time of recruitment for each participant. In brief, this human cohort consists of 101 patients of European descent that were treated with haloperidol as a monotherapy for a period of one month during the acute phase of their psychotic illness. The patients were enrolled over a 3-year period (1999–2002) at the Department of Psychiatry, Ludwig-Maximilians-University of Munich, Germany. Patients were excluded if they had a known contra-indication for treatment with haloperidol, had tardive dyskinesia previously, or severe neurological or medical disorders, organic brain diseases, pregnancy, or acute suicidal tendencies. Furthermore, the patients were excluded if they received a co-medication, such as beta blockers, or antidepressants that could possibly influence the antipsychotic treatment or its side effects. The patients were treated with haloperidol during the first phase of the illness’s episode, and then shifted to another antipsychotic treatment if there was a lack of response or if severe side effects developed. Patients were treated with haloperidol according to clinical need without any dose limitation during the acute phase of the illness. The mean dose of haloperidol was 8.76 mg/d (SD = 4.4) for day 1, 10.54 mg/d (SD = 6.0) for day 3, 10.88 mg/d (SD = 5.9) for day 7, 10.43 mg/d (SD = 6.2) for day 14, 10.95 mg/d (SD = 7.3) for day 21. As this was a naturalistic study doses were adapted to clinical requirements. Diagnosis was obtained through the Structured Clinical Interview for DSM-IV (SCID-I) interview, and psychopathological measurements were administered by two psychiatrists with highly reliable inter-rater evaluation results (k > 0.80). Haloperidol plasma levels, positive and negative syndrome scale (PANSS), ESRS, and UKU scores were assessed at baseline and on days 3, 7, 12, 21, and 28 as described [24]. Since genotyping was performed after the clinical part of the study was closed, it was not possible for any of the evaluators to be aware of the genetic results.

The alleles at 38 SNPs in *ABCB5* were genotyped on a MassARRAY platform (Sequenom) for 85 patients, which represents all of the available DNA samples obtained from the individuals in this cohort. Briefly, after a multiplex PCR and shrimp alkaline phosphatase treatment, a single base extension reaction was performed. Following the de-salting of extension products with SpectroCLEAN resin (Sequenom), samples were spotted on SpecroCHIPs GenII (Sequenom), and analyzed with a MassARRAY MALDI-TOF mass spectrometer. Allele specific extension products and the resulting genotypes were identified using Typer 3.4 Software (Sequenom). For genotyping quality assurance, the CEU HapMap Trios (Coriell Institute for Medical Research) were included and compared with the HapMap database (www.hapmap.org). Nine of the SNPs were not polymorphic in these 85 patients, and were excluded from further analysis. None of the remaining 29 SNPs violated the HWE condition, since the smallest *p*-value obtained using a traditional Chi-squared test [25] was 0.11. Of note, 26 SNPs exhibited all three possible genotypes (i.e., homozygous major allele, heterozygous and homozygous minor allele), while three SNPs had only two genotypes. For the 26 SNPs with all three possible genotypes, we used an additive model to assess the association using ANOVA model. For the three SNPs with only two observed genotypes (homozygous major allele and heterozygous), only one grouping was tested using two-sample *t*-test. For each patient, the degree of parkinsonoid toxicity was rated as 0 (absent), 1 (mild), 2 (moderate), or 3 (severe) for each time point. A patient was classified as exhibiting parkinsonoid toxicity if the score was ≥2, as not exhibiting this toxicity if the score was ≤1 at each time point. Patients were similarly rated and classified for dyskinesia and akathesia, the other two toxicities. A combined measure of HIT at each time point was defined as follows: a patient was designated as exhibiting toxicity for (i.e., 1) if any of the three toxicity measurements was ≥2, and as non-toxic (i.e., 0) if the three toxicity measurements were all ≤1. Finally, as a cumulative index of the toxic effect of haloperidol, the area-under-curve (AUC) of the combined toxicity measures for days 0, 1, 3, and 7 was calculated. This value represents the cumulative toxicity induced by haloperidol over the indicated period, and it assumes one of the following values: 0, 1, 2, 3, or 4.

## Results

### Whole Genome-Genetic Association Mapping of Susceptibility to HIT

As described in S1 Text, we analyzed NGS data to produce a 16 million SNP database with alleles covering 26 strains, including the 12 strains sequenced here, 13 strains analyzed in [11], and the reference C57BL/6 strain (S1 Fig.; S1 Table). Since our prior studies analyzed much less complex datasets with fewer SNPs and blocks, we did not know if HBCGM could analyze this far more complex (~900-fold more blocks) whole genome NGS-based dataset. However, we found that whole genome HBCGM could identify the known genetic factors for four biomedical traits (S2 Fig.; S2 Table; S3 Text). Moreover, we also found that its performance was superior to that of another genetic association method, which analyzed the correlation with a selected set of individual SNPs, especially when the inbred strains exhibited three or more distinct phenotypic responses (S3 Table).

**Fig 1:**
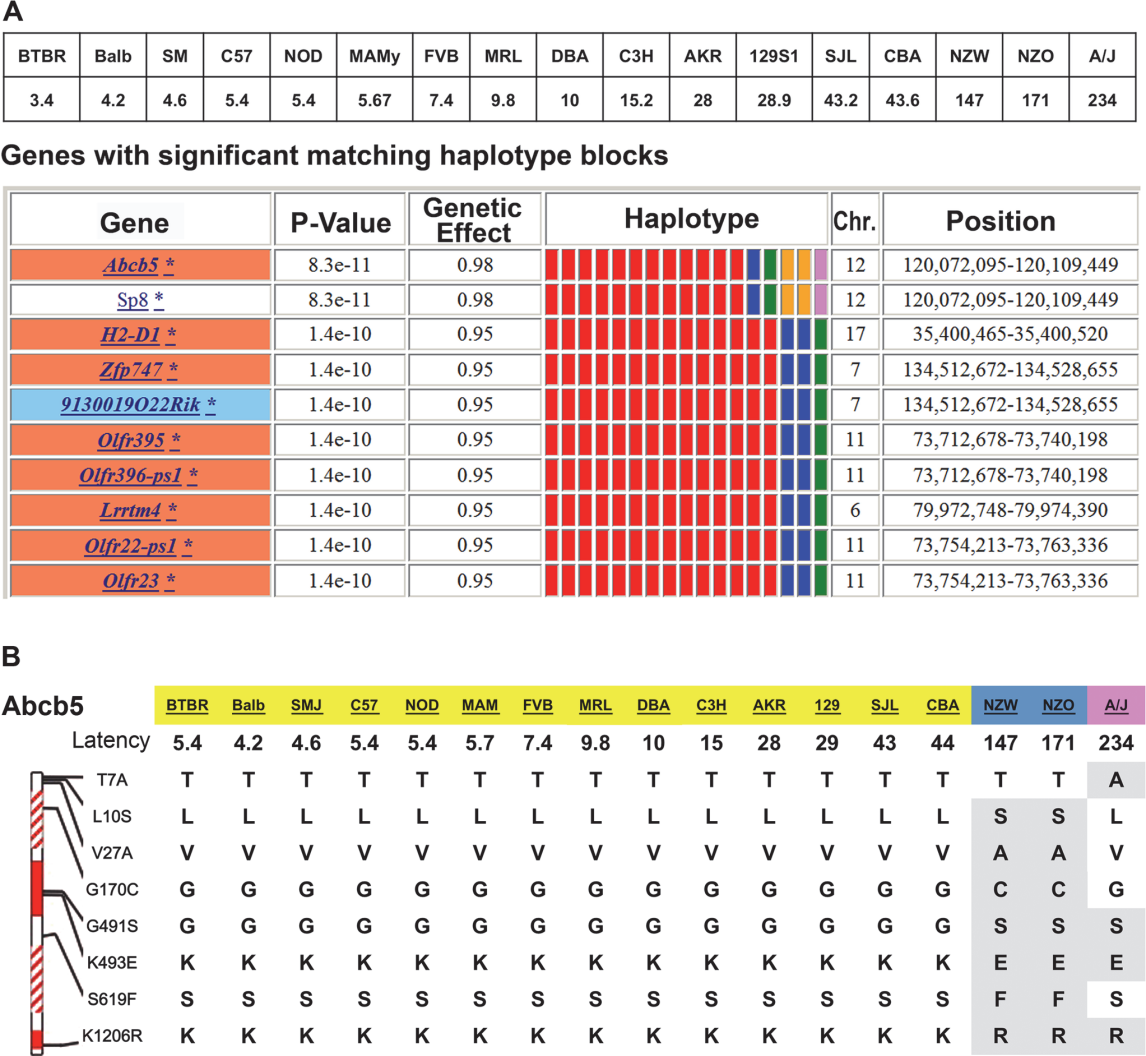
A pharmacogenetic factor for HIT. (A) The average latency measured after 30 days of haloperidol administration to 17 inbred strains is shown in the top panel. The bottom panel shows ten genes whose genetic pattern was most highly correlated with the latency data by the HBCGM program. The genes within correlated haplotype blocks are indicated by their symbol; an orange, yellow, or white background indicates whether SNPs causing a significant, minor, or no amino acid change are present, respectively. (A blue background indicates a SNP that affects a potential splice site alteration is present.) The haplotypic pattern is shown as colored rectangles that are arranged in the same order as the input data. Strains with the same colored rectangle have the same haplotype within the block. The *p*-values and genetic effect size were calculated as previously described [14]. (B) Missense SNPs in *Abcb5*. The 1,255 amino acids in Abcb5 contain two transmembrane (shaded blocks) and two ATP binding transporter domains (solid blocks). The alleles in eight missense SNPs in the 17 analyzed strains are shown, and the alleles that differ from the C57BL/6 reference strain are highlighted. The Gly170Cys is within the first transmembrane domain, and Gly491Ser and Lys493Glu are within the first transporter domain. These three SNPs divide the strains into three haplotypic groupings (each indicated by a different color) that correspond with the haloperidol-induced latency.

We then examined available data on HIT (measured after 3, 7, 30, 60, or 120 days of haloperidol treatment) for the 17 strains with available genomic sequence (S2B Table). As described in the S2B Table, our analysis of these data indicated that the prolonged latency appearing in some of the inbred strains (i) was due to haloperidol treatment; (ii) was not correlated with plasma drug levels; and (iii) had its relative magnitude in the strains maintained across all treatment periods (ranging from 3 to 120 days) analyzed. The lack of correlation between latency and plasma haloperidol level was expected, since the effect of CNS-acting drugs is dependent upon the brain (and not the plasma) drug concentration. HBCGM analysis indicated that genetic variation within *Abcb5* had the highest correlation (*p* = 8.3 × 10^−11^) with the day 30 latency values (Fig. 1A). HBCGM analysis also indicated that *Abcb5* was highly correlated with the latencies measured after 60 (rank number 3, *p*-value 2.9 × 10^−13^) or 120 (rank number 2, *p*-value 1.7 × 10^−10^) days of haloperidol treatment. Several other factors also indicated that *Abcb5* was a likely candidate gene: (i) When the robustness of the mapping results were assessed by randomly deleting sets of strains and iteratively repeating the analysis, *Abcb5* was among the top genes identified after the latency data were analyzed for treatment days 30, 60, or 120 (S4 Table). (ii) Among the 440 SNPs within or near *Abcb5*, eight missense SNPs divided the 17 strains into three haplotypic groupings with different latencies: group 1 (range: 3–44 seconds; 14 strains); group 2 (144–171 seconds; NZW, NZO); and group 3 (234 seconds, A/J). The three strains with extreme latency values share alleles at three missense SNPs that are within a nucleotide-binding region of the protein (Fig. 1B). Also, another codon changing SNP (cSNP) (rs30781912) converted a serine, which is conserved in all vertebrates, to an alanine in the strain (A/J) with the highest latency. (iii) We investigated whether *Abcb5* alleles could predict the measured latency for two inbred strains with extreme latency values (NON/ShiLtJ, 2.8 seconds; and NZL/LtJ, 211 seconds) whose genomic sequence was not yet characterized. After characterizing their *Abcb5* alleles, we found that their measured latencies were well within the range predicted by their *Abcb5* haplotype (Table 1). Lastly, genetic variation within *Abcb5* could affect susceptibility to HIT, since it is a member of the ABC-transporter gene family. Although little is known about murine *Abcb5*, its human homologue (*ABCB5)* is an energy-dependent efflux transporter that mediates the resistance to multiple drugs and chemotherapeutic agents [26–30]. Drug efflux pumps located at the luminal membrane of capillary endothelial cells in the brain, which export a drug back into the circulating blood after it has entered the brain, are major determinants of drug levels in the brain [31].

**Table 1:** *Abcb5* alleles predict haloperidol-induced latency.

SNP	NON/ShiLtJ	NZL/LtJ
T7A	T	T
L10S	L	S
V27A	V	—
G170C	G	C
G491S	G	S
K493E	K	E
S619F	S	F
K1206R	K	R
Haplotype	1	2–3
Predicted latency	0–44	147–234
Measured latency	2.75	211

**Fig 2:**
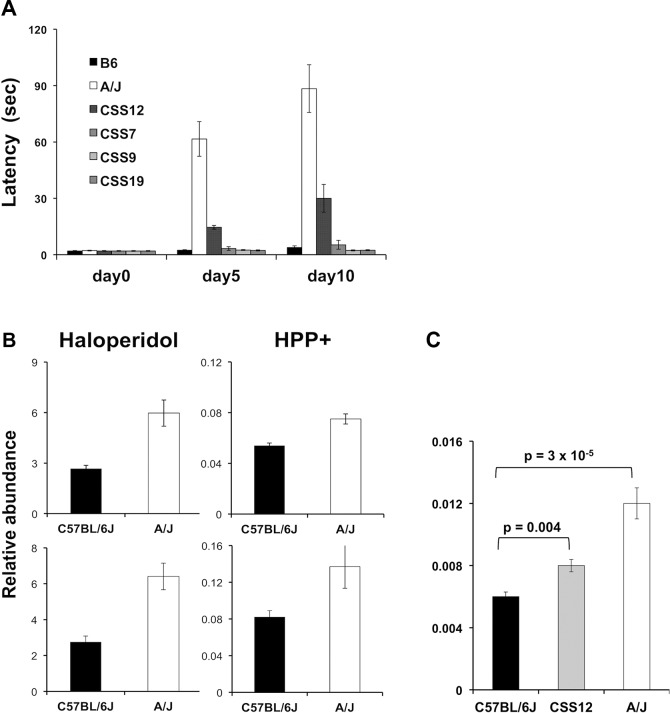
(A) HIT in C57BL/6J, A/J, and four chromosome substitution strains. The time (latency) required for a mouse to make a coordinated movement on a vertical mesh screen after treatment with haloperidol (3 mg/kg/day IP) for the indicated number of days is shown. Each bar represents the average ± SEM for three to four mice per strain. Only CSS12 mice (which have A/J alleles for every gene on Chromosome 12) had a significantly increased latency (*p* < 0.005) after 5 and 10 days of haloperidol treatment. (B) The amount of haloperidol and its metabolite (HPP^+^) in brain tissue obtained from A/J and C57BL/6 mice after 4 days of treatment with haloperidol (10 mg/kg/day IP) was assessed in two independent experiments. Each bar represents the average ± SEM of the LC/MS determined abundance (molecules per unit mass of brain tissue) for *n* = 4 mice per strain. (C) The brain level of the oxidative metabolite of haloperidol (HPP^+^) was measured after 10 days of haloperidol administration (3 mg/kg/day) to A/J (*n* = 6), C57BL/6 (*n* = 6), and Chromosome 12 substitution strain (CSS12) (*n* = 8) mice. Each bar shows the average ± standard error for the six to eight measurements for each strain, and the *p*-value is calculated using the Student’s *t* test on log2-transformed relative abundance data. Of note, CSS12 mice have a significantly higher (1.3-fold, *p* = 0.004) brain HPP^+^ level than C57BL/6J mice, but this level is below that in A/J mice.

The alleles for eight missense SNPs in *Abcb5* that were determined for two inbred strains are shown. The alleles place NON/ShiLtJ mice in haplotypic group 1, and NZL/LtJ mice in haplotypic group 2–3. The measured haloperidol-induced latencies on treatment day 30 are well within the range (based upon data in Fig. 2) predicted by their haplotype.

### Analysis of Chromosome Substitution Strains and Brain Haloperidol Levels

A series of C57BL/6J-A/J CSS have been produced where each strain is homosomic for a single specified A/J chromosome on an otherwise C57BL/6 genetic background [32]. Since A/J and C57BL/6 exhibited extreme latency values, we tested the haloperidol-induced latency in four CSS. Of these, only CSS12 (which have A/J alleles for every gene on Chromosome 12) had a significantly increased latency after 5 (6.7-fold, t value = 12.98, *p* < 0.0001, 95% CI 4.9–9.3) and 10 days (8.5-fold, t = 8.41, *p* < 0.0001, 95% CI 4.8–14.8) of haloperidol treatment (Fig. 2A). Among the 34 genes on Chromosome 12 that were correlated with susceptibility to HIT, *Abcb5* had the highest level of correlation and was the most plausible candidate based on its biological function (S5 Table).

Haloperidol is oxidatively metabolized to a pyridinium species (HPP^+^) that is present in brain tissue obtained from haloperidol-treated humans [33] or rodents [34]. HPP^+^ is a structural analog of the oxidative metabolite (MPP^+^) of a Parkinson-inducing neurotoxin N-methyl-4-phenyl-1,2,3,6-tetrahydropyridine (MPTP), and HPP^+^ has been shown to induce toxicity in dopaminergic neurons [33,35,36]. To determine if differences in brain drug (or metabolite) levels were associated with susceptibility, we measured the concentration of haloperidol and HPP^+^ in brain tissue obtained from C57BL/6J and A/J mice after treatment with haloperidol 10 mg/kg/day for 4 days. This higher dose of haloperidol was used to ensure that we could measure the brain concentrations of both haloperidol and HPP^+^, since the metabolite is present at much lower (~100-fold) concentrations than the parent drug. C57BL/6 mice treated with this haloperidol dose do not exhibit an increased latency (see below). Consistent with HIT occurring in A/J but not C57BL/6J mice, the brain levels of haloperidol (2.2-fold and 2.3-fold, t values 4.88 and 4.99, *p*-values 0.008 and 0.002, 95% CI 1.4–3.5 and 1.5–3.5, respectively) and HPP^+^ (1.4-fold and 1.7-fold, t values 4.75 and 2.39, *p*-values 0.009 and 0.05, 95% CI 1.1–1.7 and 1.0–2.6, respectively) were both higher in A/J than in C57BL/6J mice in two independently performed experiments (Fig. 2B).

We also examined HPP^+^ levels in brains obtained from A/J, C57BL/6J, and CSS12 mice after a prolonged period (10 days) of haloperidol administration (3 mg/kg/day). The brain HPP^+^ level in CSS12 mice was significantly above (1.3-fold, t = 3.22, *p* = 0.004, 95% CI 1.1–1.5) that in C57BL/6 mice, but was below that of A/J mice, which had 1.9-fold (t = 7.65, *p* = 3 × 10^−5^, 95% CI 1.6–2.2) increase in HPP^+^ relative to C57BL/6 (Fig. 2C). These results suggest that the differential strain susceptibility to HIT could result from differences in Abcb5-regulated drug transport, and that an A/J Chromosome 12 on an otherwise C57BL/6 genetic background increases brain HPP^+^ levels. The correlation between the relative brain abundance of HPP^+^ in A/J, C57BL/6, and CSS12 mice and their haloperidol-induced latencies (Fig. 2A) is consistent with the other studies [33,35,36] indicating that HPP^+^ is causative of HIT.

### Desorption Electrospray Ionization-Mass Spectrometry Imaging of Haloperidol Abundance in Brain Sections

**Fig 3:**
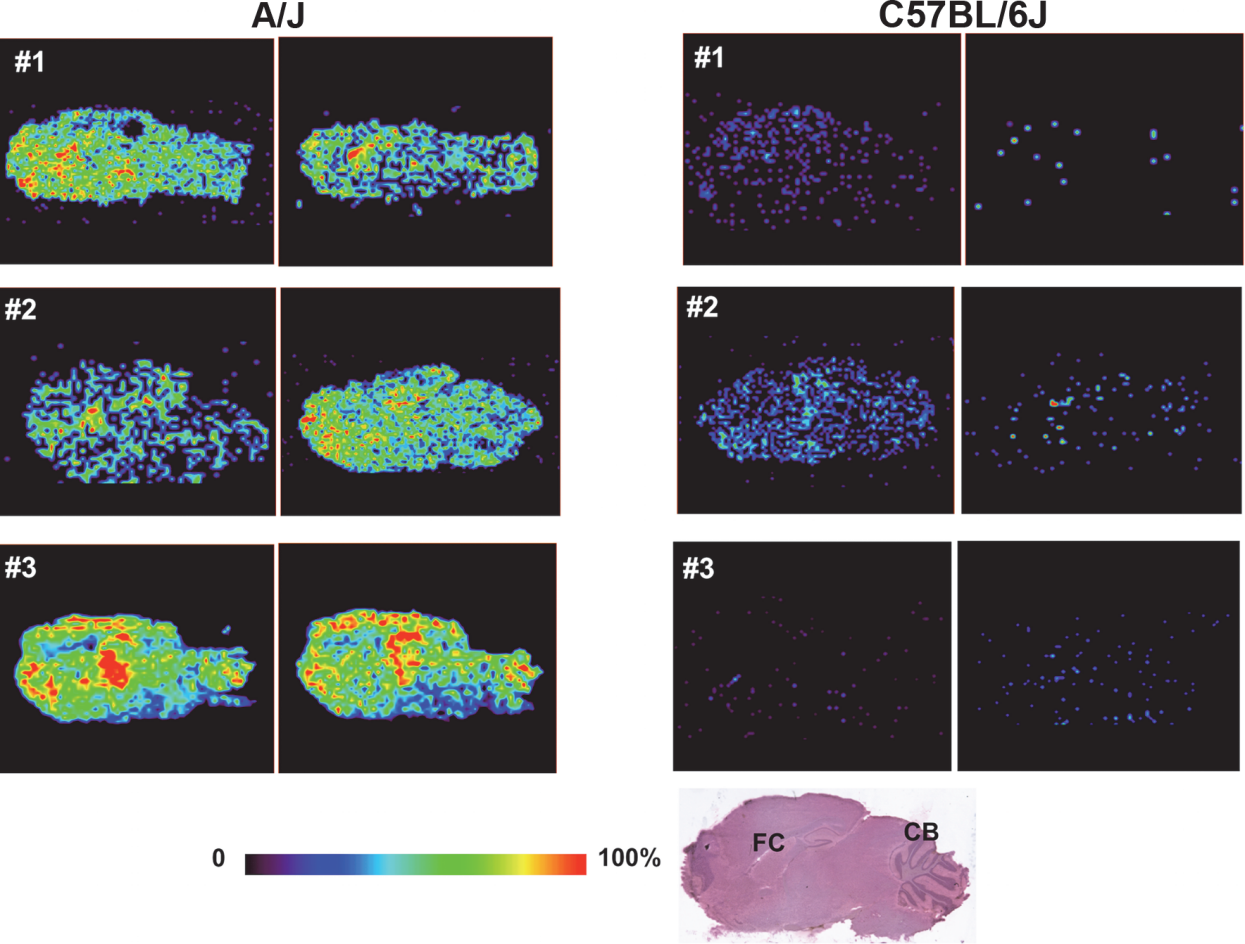
DESI-MSI of haloperidol in brain tissue. A/J and C57BL/6J mice (*n* = 3 per strain) were treated with haloperidol (10 mg/kg/day IP) for 5 days. Two sagittal sections, which were separated by a distance of 120 μm, were prepared the same brain region of haloperidol-treated mice. The amount of haloperidol was analyzed DESI-MSI, and the relative abundance of haloperidol is indicated by the color shown in the scale bar. The HE-stained brain section shown at the bottom shows the sagittal plane analyzed, and the location of the frontal cortex (FC) and cerebellum (CB) are indicated for orientation. Haloperidol was diffusely present in the brain tissue obtained from both strains. Although the amount of haloperidol in brain tissue varied among different mice, A/J brain tissue had remarkably increased amount of haloperidol relative to C57BL/6J brain tissue.

In DESI-MSI, thin tissue sections are bombarded with solvent microdroplets to dissolve hundreds of chemicals from the sample surface; the secondary microdroplets formed enter a mass spectrometer, and are analyzed for their chemical content. A movable stage enables multiple pixels (in a row by column matrix) encompassing the entire thin section to be sampled, which generates a detailed 2-D chemical map of the distribution of molecules within the sample surface. The relative amount of haloperidol in each region was analyzed by this method to produce a 2-D ion image of its relative abundance throughout the entire brain section. We analyzed independently prepared sagittal brain sections (two per mouse) from A/J and C57BL/6 mice (three per strain) after treatment with haloperidol (10 mg/kg/d IP) for 5 days. This analysis revealed that haloperidol was diffusely distributed throughout all brain regions, but the amount of haloperidol in A/J brain tissue was consistently greater (~6-fold) than in C57BL/6 brain tissue (Fig. 3). Because of its low abundance, we could not detect the oxidative metabolite by this method. The inter-strain differences indicated by DESI-MSI were greater than those identified by quantitative MS analysis of extracted whole brain. This difference could be attributed to the fact that DESI-MSI was performed on tissue sections in a specific sagittal plane, which does not account for the abundance of haloperidol in other brain regions that were sampled by quantitative MS analysis of extracted whole brain. Nevertheless, direct analysis of brain tissue and DESI-MS imaging both clearly show that haloperidol was more abundant in A/J mouse brain.

### *Abcb5* mRNA Is Expressed near Brain Capillaries and by Purkinje Cells

**Fig 4:**
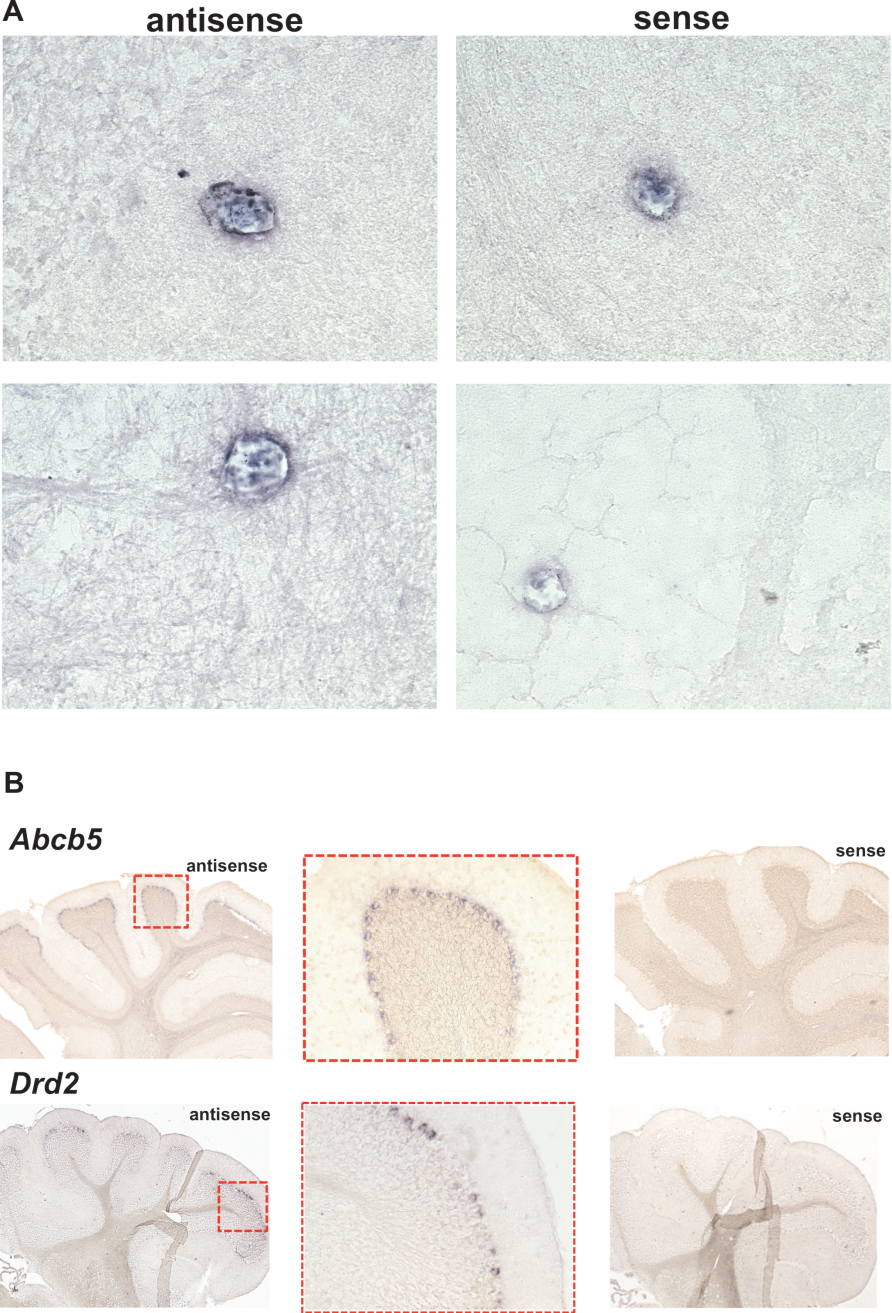
*Abcb5* mRNA expression in mouse brain. In situ hybridization was performed using anti-sense probes for *Abcb5* and *Drd2* mRNA on C57BL/6 mouse brain sections. For each anti-sense probe, negative-control hybridizations were also performed using the corresponding sense RNA probe on adjacent tissue sections. (A) *Abcb5* mRNA was specifically expressed in the perivascular regions, as shown in two separate views of cortical and cerebellar regions. (B) Purkinje cells in cerebellum express *Abcb5* mRNA, and these cells also express the dopamine receptor D2 (*Drd2*) that is bound by haloperidol. The left and right images are shown at 4× magnification, and magnified views (20×) of the boxed regions are shown in the center image.

Since we did not know if *Abcb5* is expressed in brain, in situ hybridization studies were performed on tissue sections from mouse brain. The hybridizations were performed using an anti-sense probe that hybridizes to *Abcb5* mRNA, and with an *Abcb5* sense probe that serves as a negative control. *Abcb5* mRNA expression was observed in the perivascular region of brain capillaries (Fig. 4A). Since this is the anatomic location of the blood-brain barrier function, this expression pattern is consistent with *Abcb5* having a role in regulating brain haloperidol levels. Of importance, Purkinje cells in the cerebellum also expressed *Abcb5* mRNA; and these cells also expressed dopamine receptor D2 (*Drd2*) mRNA, which encodes the receptor that binds haloperidol (Fig. 4B). Interestingly, *Abcb5* mRNA was not detected in the substantia nigra (S3 Fig.). A low level of *Abcb5* mRNA expression was also detected in regions of the hippocampus and frontal cortex (S4 Fig.), but not by the *Drd2*-positive cells in the substantia nigra (S3 Fig.).

### *Abcb5* Knockout Mice Have a Prolonged Haloperidol-Induced Latency and Increased Brain Haloperidol Levels

**Fig 5:**
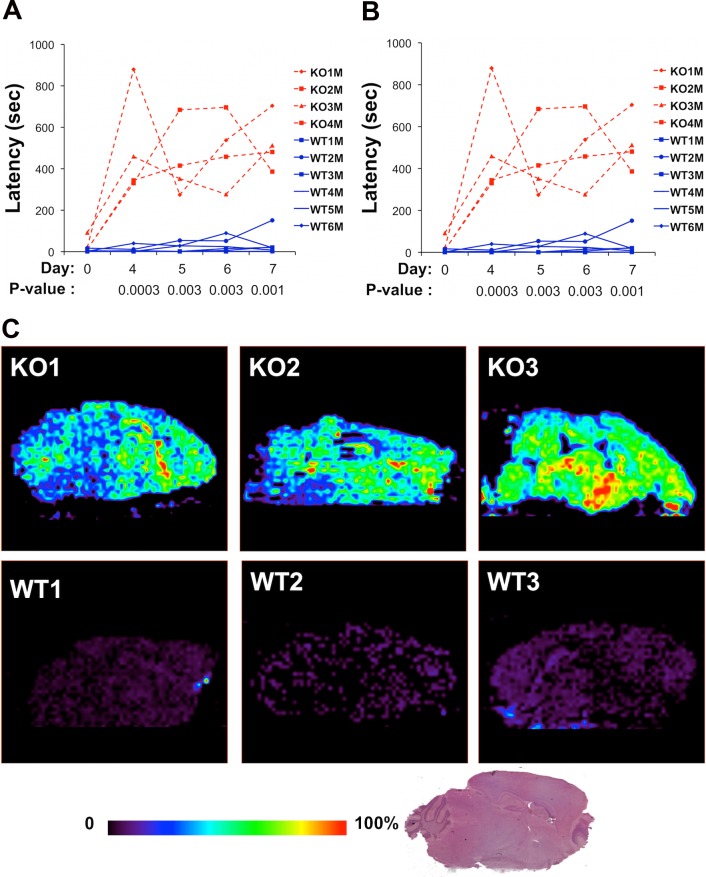
(A, B) *Abcb5* knockout mice have a prolonged haloperidol-induced latency. In two independent experiments shown in (A) and (B), *Abcb5* knockout mice and wild-type littermates (gender indicated by male or female) were treated with haloperidol (10 mg/kg IP) for 7 days, and the haloperidol-induced latency was measured on the indicated days. In both experiments, the latencies in *Abcb5* knockout mice were significantly prolonged relative to wild-type mice after 4, 5, 6, and 7 days of haloperidol treatment. The *p*-values comparing the latencies measured in the *Abcb5* knockout and wild-type groups are shown for each treatment day. (C) DESI-MSI of haloperidol in brain tissues of *Abcb5* knockout (*n* = 3) and wild-type littermate (*n* = 3) mice. The mice were treated with haloperidol (10mg/kg/day IP) for 4 days, and brain tissues were collected 4 hours after the last treatment. The amount of haloperidol was analyzed by DESI-MSI on 20 μm brain sections, and the relative abundance of haloperidol is indicated by the color shown in the scale bar. The HE-stained brain section shown at the bottom shows the sagittal plane analyzed. *Abcb5* knockout mice have a 7-fold increased brain haloperidol level relative to wild-type littermates (*p* = 0.02).

To more precisely determine whether *Abcb5* plays a role in HIT, we measured the latencies in homozygous *Abcb5* knockout mice (on a C57BL/6 background) before and after treatment with haloperidol (10 mg/kg/day IP). Before drug treatment, there was no difference in the latency of *Abcb5* knockout mice relative to wild-type littermates (t = 1.54, *p* = 0.185) (Fig. 5A). Although there were only marginal differences on treatment day 4 (t = 2.15, *p* = 0.05), the latencies in *Abcb5* knockout mice were significantly prolonged after 5 (10.1-fold, t = 3.09, *p*-value = 0.03, 95% CI 1.5–69.2), 6 (13.6-fold, t = 4.79, *p*-value = 0.005, 95% CI 3.5–55.1), and 7 (50.3-fold, t = 7.91, *p*-value = 0.0005, 95% CI 14.1–179.6) days of haloperidol treatment relative to wild-type mice (Fig. 5). In a second independently performed experiment, haloperidol treatment induced a markedly prolonged latency in *Abcb5* knockout mice (Fig. 5B). In contrast, the latencies measured in wild-type littermates did not increase with haloperidol treatment and were comparable to those of C57BL/6 mice. Furthermore, DESI-MSI revealed that the amount of haloperidol in brain tissue obtained from *Abcb5* knockout mice (*n* = 3, 6,282 ± 2,279) was 6.8-fold higher than in wild-type littermates (*n* = 3, 935 ± 200, *p* = 0.02) (Fig. 5C). The prolonged latency and increased brain haloperidol levels observed in *Abcb5* knockout mice confirms that *Abcb5* affects susceptibility to HIT by increasing brain haloperidol levels.

### Human *ABCB5* Alleles Associate with Haloperidol Toxicity

**Fig 6:**
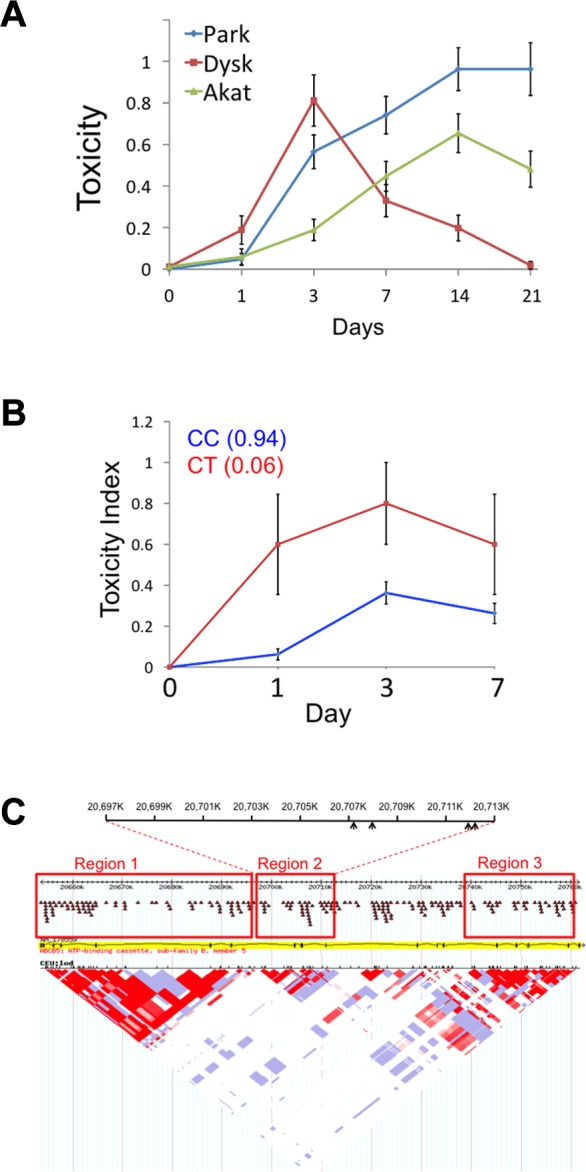
Human *ABCB5* alleles and HIT. (A) The average of three toxicity measurements (± SEM) for 85 patients determined after the indicated time of haloperidol treatment is shown. Of note, the average Dyskinesia score peaked at 3 days post treatment and then declined, while the two other toxicity measurements (akathesia and parkinsonoid) increased with time after treatment until day 14. For this analysis, the toxicity measurement data for days 0, 1, 3, 7, 14, and 21 were available for 85, 85, 85, 85, 81, and 54, respectively, of the 85 patients in this cohort. (B) The graph shows the combined toxicity measurement (*y*-axis) relative to the time (*x*-axis) for *ABCB5* SNP rs17143212. The average combined toxicity measurement was calculated for each genotype at each SNP, and then plotted (± SEM) according to the colors shown in the legend. (C) The LD map for genetic variants in *ABCB5*, which was compiled using data obtained from the International HapMap project (http://hapmap.ncbi.nlm.nih.gov/). The three regions where the alleles have a high level of LD are enclosed in boxes. Region 2 contains a cluster of four SNPs with alleles that were associated with HIT, and arrows in the diagram at the top of the figure indicate their relative positions.

A human cohort with 85 patients of European descent was treated with haloperidol as a monotherapy for one month during the acute phase of their psychotic illness, and their response to therapy and the appearance of HIT was assessed at baseline and after 1, 3, 7, 14, and 21 days of treatment as described [24]. Their clinical and demographic characteristics are described in Table 2. We investigated whether *ABCB5* SNP alleles were associated with the appearance of the three types of symptoms of HIT (Parkinsonoid, Dyskinesia, and Akathisia scores) that were measured in this cohort. Of the 29 SNPs analyzed: six were codon changing (cSNPs), three were in the 5′ UTR, and the others were intronic (Table 3). We first examined the effect that treatment duration had on the incidence of each of the three types of HIT toxicities throughout the course of treatment (S6 Table). The incidence of akathesia and parkinsonoid features increased through day 14, while dyskinesia decreased after day 3 (Fig. 6A), which may be due to fact that the onset of the other toxicities masked the dyskinesia. Therefore, we analyzed a composite index of all three symptoms of HIT, since our mouse genetic analysis identified a genetic factor that alters the brain haloperidol concentration, which will affect susceptibility to all of these symptoms. In examining the allelic associations, we found a clear time-dependent allelic effect on HIT that was maximal on days 1, 3, and 7 (Figs. 6B and S5). Therefore, it is likely that the protective allelic effect would dissipate after prolonged dosing owing to haloperidol (or metabolite) accumulation, but individuals with a protective *ABCB5* allele would exhibit less toxicity during the early treatment period (i.e., days 3–7).

**Table 2:** Demographic and clinical data for the 85 patients.

Variable	Results
Age (y) (mean SD)	35.4 11.7 (Range: 18–64)
Sex	M = 45 (53%); F = 40 (47%)
Diagnosis	Schizophrenia, paranoid type = 46 (54.1%)
	Schizophrenia, undifferentiated type = 2 (2.4%)
	Schizophrenia, residual type = 2 (2.4%)
	Schizophrenia, catatonic type = 4 (4.7%)
	Schizophrenia, disorganized type = 3 (3.5%)
	Schizoaffective disorder = 15 (17.6%)
	Brief psychotic disorder = 7 (8.2%)
	Delusional disorder = 1 (1.2%)
	Schizophreniform disorder = 5 (5.9%)
Co-medication (till day 7)	Levomepromazin/ppromethazin = 52 (61.2%)
	Cholinergic (biperiden/amitriptylin) = 26 (30.6%)
	β-blocking agents = 8 (9.4%)
	Gabaergic agents and benzodiazepine = 45 (52.9%)
Comorbid illness	Phobia = 2 (2.4%)
	Alcoholism = 3 (3.5%)
	Depression = 3 (3.5%)
	Suicide attempt = 5 (5.8%)
Reason for dropout	
Day 14	4 discharged after good recovery (4.7%)
Day 21	1 discharged after good recovery (1.1%)
	7 switched to other meds after good recovery (8.2%)
	7 switched after good recovery but with side effects (8.2%)
	3 without recovery (3.5%)
	9 intolerable side effects (10.5%)

**Table 3:** Genetic association results for alleles at 29 SNPs with the area under curve calculated by summing the combined toxicity measures on days 0–7.

SNP ID	Position	Annotation	Region	MAF	AUC	Permutation *p*-Value
rs2106562	20655268	5′UTR		0.35	0.044	0.523
rs73076550	20655392	5′UTR		0.11	0.272	0.988
rs78414512	20668277	INTRON		0.16	0.005	0.086
rs17143212	20682884	T131I	1	0.03	0.002	0.034
rs2893006	20687181	5′UTR / Synonymous	1	0.24	0.308	0.995
rs75784515	20687332	INTRON	1	0.24	0.287	0.992
rs61732039	20687604	D370G	1	0.14	0.03	0.402
rs3213622	20690885	INTRON	1	0.34	0.434	0.999
rs34603556	20691047	Alternative start codon / M446T	1	0.24	0.308	0.995
rs61227829	20691219	Synonymous	1	0.15	0.038	0.459
rs17816709	20691860	INTRON	1	0.24	0.287	0.992
rs2301641	20698270	K115E / K560E	1	0.36	0.588	1
rs6952128	20700361	INTRON	1	0.36	0.463	1
rs17143258	20726621	INTRON	1	0.18	0.531	1
rs12700229	20729931	INTRON	2	0.26	0.719	1
rs2190409	20732020	INTRON	2	0.19	0.961	1
rs17218211	20740648	INTRON	2	0.21	0.011	0.195
rs10488579	20741343	INTRON	2	0.24	0.021	0.316
rs17218839	20745418	INTRON	2	0.26	0.026	0.361
rs2108259	20745756	INTRON	2	0.26	0.024	0.338
rs62453384	20762646	G365V / G810V		0.38	0.478	1
rs62453385	20762937	INTRON		0.38	0.478	1
rs6461513	20763907	INTRON		0.32	0.031	0.402
rs4719626	20768794	INTRON		0.26	0.102	0.798
rs6960186	20773995	INTRON	3	0.31	0.478	1
rs6461515	20778646	E970K / E525K / E137K	3	0.19	0.117	0.853
rs6461516	20778773	INTRON	3	0.17	0.189	0.961
rs6461517	20787273	INTRON	3	0.38	0.706	1
rs1015646	20793720	INTRON	3	0.39	0.681	1

The SNP ID, genomic position (based on NCBI Build 37), minor allele frequency (MAF), and the *p*-value (uncorrected for multiple testing) for the allelic association with the combined toxicity measure are shown. The annotation indicates the location of the SNP (intron, 5′ or 3′ UTR), and the amino acid residue altered by the SNP (because of alternative *ABCB5* transcripts, more than one location is indicated for some SNPs). All *p*-values < 0.05 are in bold. To account for multiple testing a permutation test was performed for each SNP, and the *p*-value is shown in a separate column. Of note, rs17143212 is a missense SNP (Thr131ILE) that it is the only SNP with a significant *p*-value in the permutation test. This indicates that its association with the area under the curve (AUC) remains significant even after accounting for the fact that 29 SNPs were simultaneously tested.

Since each of these three different toxicities could all be caused by an increased drug (or drug metabolite) concentration in the brain, we examined whether *ABCB5* alleles were associated with a combined toxicity index, which indicated whether a patient manifested any of the three measured toxicities on a given day. To reduce the number of hypotheses that were tested simultaneously, the area under the curve (AUC) for the cumulative toxicity measurements on days 0 to 7 was calculated, and its association with each of the 29 SNPs was tested. (A truncated dataset was used because many of the values for day 14 and 21 were not available for many patients.) Remarkably, a SNP (rs17143212) that was a missense SNP (Thr131Ile) had a particularly strong association (raw *p* = 0.002) with the toxicity index, and a total of ten (out of 29) SNPs showed a plausible association (with raw *p* < 0.05) with the toxicity index (Table 2). Among these SNPs, four were consecutive SNPs within the second identified region of linkage disequilibrium in the *ABCB5* gene (Fig. 6C). The probability that ten SNPs would randomly have these *p*-values (< 0.05) is <8 × 10^−7^, indicating that it is highly unlikely that all of these associations were false positives resulting from a random match. Since the AUC values didn’t follow a Gaussian distribution and the sample size may not be large enough, the resulting *p*-values from the ANOVA models could be biased. Therefore, we performed a permutation test, which accounts for multiple testing and makes no assumption about the data distribution, to rigorously assess the statistical significance of each association. To do this, the combined toxicity value for the 85 patients was permutated, while their genotypes remained unchanged; this enabled the linkage disequilibrium among the SNPs to be retained. The distribution of the minimum *p*-value for the 29 SNPs was evaluated by performing this permutation experiment 1,000 times. The observed raw *p*-value for each SNP was then compared to this distribution to obtain the true *p*-value for that SNP under the null hypothesis that this region was not associated with HIT. Strikingly, rs17143212, which caused a significant (131Thr → ILE) amino acid change in the predicted protein, still exhibited a significant association (permutation test *p*-value = 0.034) after the permutation test. The four consecutive SNPs that had significant raw *p*-values didn’t have significant permutation test *p*-values. However, if additional patients were available for analysis, we would be able to better assess the significance of their associations. Nevertheless, at least one human *ABCB5* SNP shows a significant association (after correction for multiple testing) with a combined measure of HIT.

## Discussion

**Fig 7:**
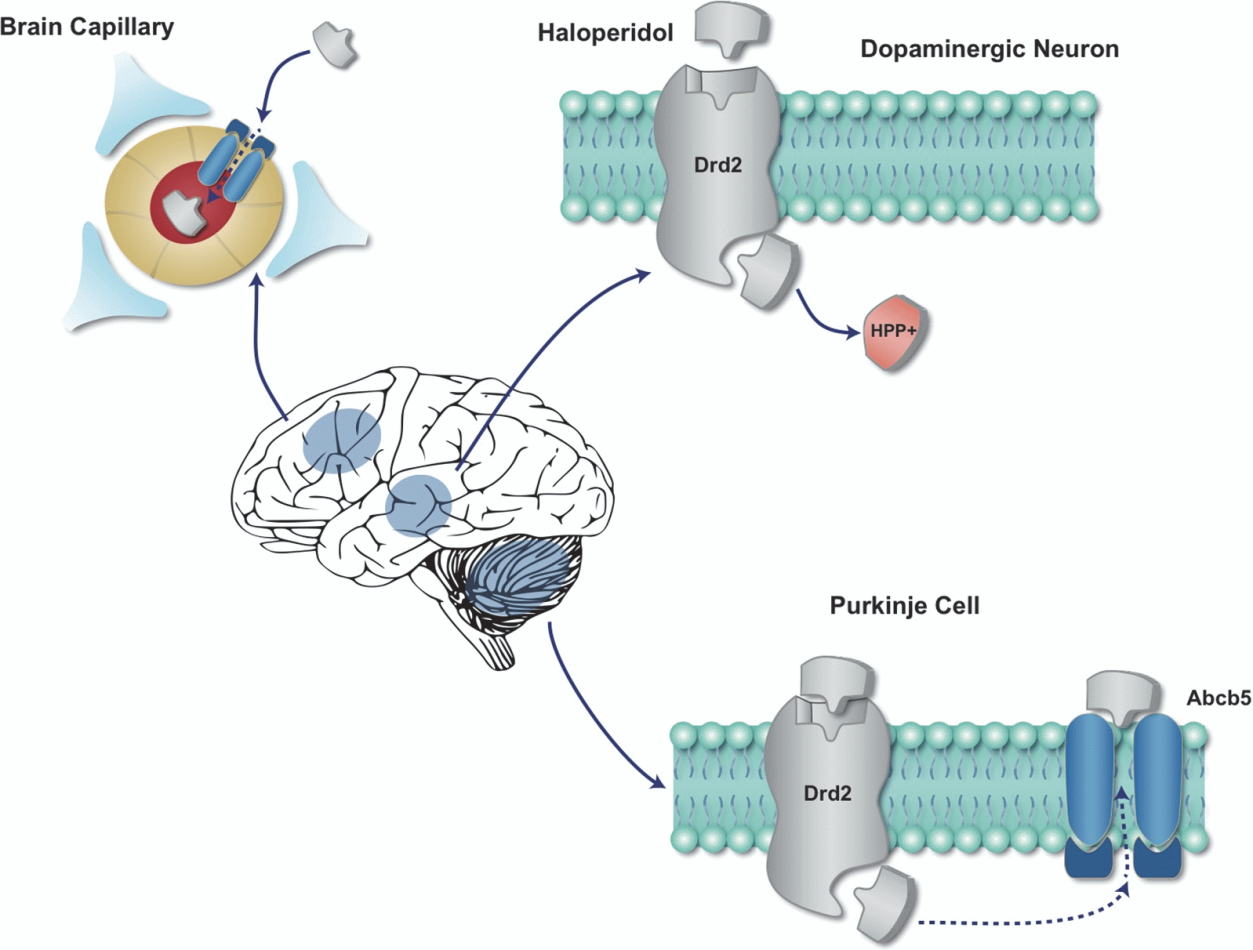
A diagram of the effect of Abcb5-mediated haloperidol transport on HIT. Abcb5 is expressed in brain capillaries and by cerebellar Purkinje cells. Haloperidol will enter the brain due to its intrinsic properties, but vectorial transport by perivascular Abcb5 will reduce the brain concentration of haloperidol. An individual with alleles that reduce Abcb5 activity are more susceptible to HIT because they will have an increased brain haloperidol concentration. Cerebellar Purkinje cells and dopaminergic neurons in the substantia nigra both express the Drd2 receptor that is bound by haloperidol. In Drd2-expressing dopaminergic neurons haloperidol is converted to its oxidative metabolite (HPP^+^), which inhibits mitochondrial energy generation and causes the characteristic Parkinsonian-like symptoms to develop. Although Purkinje cells express Drd2, they also express Abcb5, which exports of haloperidol from these cells and protects them from HIT.

This study identifies murine *Abcb5* alleles as pharmacogenetic factors affecting susceptibility to HIT. Because Abcb5 is expressed in brain capillaries, it can function as part of the blood-brain barrier to regulate the level of drugs in the brain [37]. This function is consistent with the measured differences in the brain levels of haloperidol and its metabolite (HPP^+^) in inbred and chromosome substitution strains. These findings suggest a new model for the genetic mechanisms underlying inter-individual and cell type-specific differences in susceptibility to haloperidol-induced CNS toxicity (Fig. 7).

After haloperidol enters the brain (because of its lipophilicity or other intrinsic properties), the brain concentrations of haloperidol and its toxic metabolite will be affected by Abcb5 function at the brain-blood barrier. Thus, a strain with an *Abcb5* allele with reduced transporter function is more susceptible to HIT because of the increased haloperidol concentration in the brain. Like the causative toxins (MPTP, rotenone) used in the other animal models of toxin-induced Parkinson’s disease [38–41], the oxidative metabolite of haloperidol (HPP^+^) is a potent inhibitor of NADH-linked mitochondrial respiration [36]. Brain enzymes convert the Parkinsonian neurotoxin MPTP to its toxic metabolite [42–44], which is concentrated in cells with a high affinity dopamine uptake system [41,45], which causes the Parkinsonian-like symptoms to develop. HPP+ is a more potent inhibitor of mitochondrial electron transport than the oxidative metabolite of MPTP [36]. The toxicity of paraquat, which is another neurotoxin that selectively damages dopaminergic neurons, is also dependent upon dopamine transporter activity [46]. Thus, we propose that haloperidol in Drd2-expressing dopaminergic neurons is converted to its oxidative metabolite HPP^+^ by the drug metabolizing enzymes present in these neurons [47,48]; and the mitochondrial toxicity induced by this metabolite produces the Parkinsonian-like symptoms.

Of interest, we found that Abcb5 and Drd2 were also expressed in cerebellar Purkinje cells. Given their large size, high metabolic requirements, and Drd2 expression, Purkinje cells should also be affected by HIT, since they are particularly susceptible to toxin-induced injury [49]. However, the symptoms of HIT observed in mice and humans are predominantly extrapyramidal and do not have a substantial cerebellar component. Why does HIT produce Parkinsonian symptoms, but not cerebellar toxicity? We believe that Abcb5 expression by Purkinje cells protects them from HIT by their ability to transport haloperidol out of the cells. In contrast, haloperidol-targeted neurons in the substantia nigra, which lack Abcb5 expression, will be susceptible to HIT (Fig. 7).

Although a relatively small cohort of patients who were treated with haloperidol was evaluated here, a human *ABCB5* SNP, which causes a significant amino acid change in the protein, was also associated with susceptibility to HIT. However, the human allelic affect is exerted during the early period of drug treatment. The time-dependent effect observed in humans may be caused by the fact that continued haloperidol treatment may overwhelm the ability of the protective allele to clear the toxic metabolite. Despite some evidence for familial clustering [50] and scattered reports (from studies that evaluated a small number of patients) of candidate gene associations [51,52], we did not have any solid information about human pharmacogenetic factors for HIT. A recently completed, large human GWAS did not reveal any significant associations with antipsychotic drug-induced toxicities [53]. However, this study did not perform the detailed analysis of HIT that was performed here. Our results suggest that an examination of the time dependence of individual and the aggregation of cumulative toxicity measurements may be required to identify pharmacogenetic factors for antipsychotic agents. Like the murine gene, human *ABCB5* is also highly polymorphic; alleles at ten codon changing SNPs (cSNPs) are predicted to affect human ABCB5 transporter function, including two (Iso648Thre, Glu679Lys) within the nucleotide-binding region [54]. So, it is certainly possible that analysis of a larger cohort could identify other human *ABCB5* alleles that also affect ABCB5 function, and possibly susceptibility to drug toxicity. It is also possible that analysis of a larger human cohort could reveal SNP alleles in other genes that also affect susceptibility to HIT. Although haloperidol is a first-generation antipsychotic agent, the more commonly used second-generation antipsychotics (aripiprazole, clozapine, olanzapine, risperidone) are also associated with a significant incidence of EPS-related toxicities, which is higher than was originally appreciated [55]. It would be of interest to determine whether genetic variation in *ABCB5*, or other efflux transporters, could affect susceptibility to their toxicity. Simulation studies have convincingly demonstrated that using pharmacogenetic information for drug selection [56] or dose adjustment [57] could reduce drug toxicity. Characterization of genetic factors affecting the toxicity caused by haloperidol (or second-generation antipsychotics) could lead to pharmacogenetic strategies that would improve the outcome for patients taking these drugs.

Lastly, analysis of chromosomal substitution strains indicated that the presence of A/J alleles on Chromosome 12 could partially induce susceptibility to HIT. However, the magnitude of the haloperidol-induced latency and the brain HPP^+^ levels in CSS12 mice were significantly below those of A/J mice. Thus, other genetic factors could also affect susceptibility to HIT in mice, which raises the possibility that there are other human pharmacogenetic factors to be found.

## Conclusions

These findings indicate that Abcb5 is a component of the blood-brain barrier in mice and suggest that genetic variants in this gene underlie, at least in part, the differences in susceptibility to haloperidol-induced toxicity among inbred mice strains. Moreover, the human genetic association study indicates that a specific *ABCB5* allele also affects the susceptibility of people to haloperidol-induced toxicity.

## Supporting Information

S1 Checklist ARRIVE checklist. (DOCX)

S2 Checklist STROBE statement. (DOCX)

S1 Fig The effect of incorporating SNP data obtained from additional strains on the haplotype map. (A) The number of total (left panel) and added (right panel) SNPs identified after analysis of the genomic sequence obtained from the indicated number of additional strains. Beginning with the 12.6 M SNPs present in 14 strains, additional SNPs were identified by analyzing the sequence of 11 additional strains in a random order. This procedure was repeated 50 times, and the order of the added strains was varied. The left panel shows the total number of SNPs ± SEM from for each additional strain; while the right panel shows the number of added SNPs ± SEM for each added strain. Of note, the total number of SNPs increased significantly for each added strain. However, the number of new SNPs identified per additional strain analyzed decreased in an exponential fashion (blue line) as additional strains were evaluated. (B) The total number (± SEM) of haplotype blocks identified after inclusion of SNPs that were identified by analysis of the NGS sequence data obtained from the 15th through 25th strains that were analyzed is shown. These numbers were determined using the simulation described in (A). (C) The total numbers of additional haplotype blocks (± SEM) that were identified after incorporation of SNP data from the indicated number of additional analyzed strains are shown as diamonds. The numbers of added blocks (± SEM) that were produced as a result of new SNPs present in the additional analyzed strains are shown as circles. Of note, 30%–53% of new haplotype blocks are produced from newly identified SNPs, while the remainder are produced by new recombinations occurring in regions with previously known SNPs. (D) The average size of a haplotype block (± SEM) decreases as the additional allelic data are incorporated into the genetic map. (E) A density plot of the quality (QUAL) score for the SNP calls, which were determined by analysis of the sequence of each indicated strain using Samtools. The QUAL score reflects the likelihood that there is a genetic variant at each identified position. One criterion for inclusion of a SNP in the database was a QUAL score ≥ 50. Since the SJL strain had 55-fold whole genome sequence coverage, it is noteworthy that 68% of SJL SNPs have a QUAL score > 150; while the other strains, with 20- to 31-fold sequence coverage, had only ~50%–60% of their SNPs above the same cutoff. However, regardless of the extent of sequence coverage, ~87% of the SNP calls for all strains are above the desired cutoff of 50.
(TIF)

S2 Fig The input phenotypic data and the HBCGM results for the aryl hydrocarbon response and macrophage susceptibility to *Bacillus anthracis* lethal toxin (A), the major histocompatibility complex (MHC) H2 response (B), albino skin type (C), and survival after fungal infection (E) are shown. The *p*-value, and Genetic Effect Size were determined as previously described [8]. The genes within correlated haplotype blocks are indicated by their symbol; a blue, orange, yellow, or white background indicates whether SNPs altering a splice site, or causing a significant, minor, or no amino acid change within the predicted protein sequence are present, respectively. The haplotypic pattern is shown as colored rectangles arranged in the same order as the input data. Strains with the same colored rectangle have the same haplotype within the block at the indicated chromosome and position. The presence (*y*) or absence (*n*) of albinism in each strain is indicated in (C). (D) The association between SNP alleles in two candidate genes and albino status are shown for four additional strains: one strain (P/J) is non-albino (green) and three are albino (red). The alleles for each SNP are indicated by letter and by box color. Only the Cys103Ser alleles in the *Tyr* gene segregate with the albino status of these four strains.
(TIF)

S3 Fig DRD2^+^ cells in the substantia nigra do not express Abcb5. In situ hybridizations were performed using anti-sense probes for *Abcb5* and *Drd2*. The dashed box region shows the substantia nigra region. The images of sagittal brain sections were obtained from a C57BL/6 mouse, and are shown at 1× magnification.
(TIFF)

S4 Fig *Abcb5* mRNA is expressed in the hippocampus (A) and in frontal cortex (B). In situ hybridization was performed using anti-sense probes for *Abcb5* on C57BL/6 mouse brain sections. As a negative-control, hybridizations were also performed using the sense probe on adjacent tissue sections. *Abcb5* mRNA was expressed in a linear pattern within the hippocampus (arrows). In addition, Abcb5 was also faintly expressed in the frontal cortex. The images are shown at 4× magnification, and a magnified view (20×) of the boxed region in (A) is shown in the center image.
(TIFF)

S5 Fig The graphs show the combined toxicity measurement (*y*-axis) relative to the time (*x*-axis) for each of the four consecutive *ABCB5* SNPs located between 20.740 and 20.746 MB. The average combined toxicity measurement was calculated for each genotype at each SNP, and then plotted according to the colors shown in the legend.
(TIFF)

S1 Table The sequence coverage and SNP variants identified by analysis of NGS data for 26 mouse genomes. The amount of genomic sequence (Gb), fold coverage, numbers of SNPs, or indels relative to the C57BL/6 reference sequence (or uniquely present in each strain) are shown. The sequence data for the first 12 strains was obtained at Stanford, C57BL6 is the reference sequence, and Keane and colleagues [1] produced the sequence data for the other 13 strains. The sequence coverage and SNP variants identified by analysis of NGS data for 26 mouse genomes. The amount of genomic sequence (Gb), fold coverage, numbers of SNPs, or indels relative to the C57BL/6 reference sequence (or uniquely present in each strain) are shown.
(DOCX)

S2 Table (A) The phenotypic data used for HBCGM of five traits. The aryl hydrocarbon response (AHR), major histocompatibility complex (MHC) H2 haplotypes (MHC H2), litter size (MPD: 31408), and macrophage susceptibility to the *Bacillus anthracis* lethal toxin (Anthrax MPD: 1501) were obtained from [8] and from the Mouse Phenome Database (http://phenome.jax.org/). Since the MHC H2 haplotypes for NOD/ShiLtJ (g7), SJL/J (s), and SM/J (v) are unique and each is distinct from that of the other 18 strains; they were not used for the HBCGM results shown in this paper. We can include no more than five different phenotypes in a mapping experiment since haplotype blocks with up to five haplotypes are analyzed. The albino status (Albino) was obtained from the Jackson Laboratory description of mouse strains (http://www.jax.org/). The *Candida albicans* (*C*. *albicans*) survival data was obtained from [11]. (B) The phenotypic dataset used for analysis of haloperidol-induced latency. The measured haloperidol-induced latencies (seconds) on days 0 (MPD: 39407), 3 (MPD: 39408), 7 (MPD: 39409), 30 (MPD: 39410), 60 (MPD: 39411), and 120 (MPD: 39445); and the plasma haloperidol levels on day 30 (MPD: 39403) were obtained from the Mouse Phenome Database (MPD) (http://phenome.jax.org). The number of mice examined, average measurement, and the standard deviation are shown for each of the 16 indicated strains.
(DOCX)

S3 Table The results obtained using the EMMA method [5] for evaluating the phenotypic data for five traits. Genetic variation within the indicated causative genes was among the most highly correlated candidates identified by HBCGM. After EMMA analyzed the same phenotypic data, its results were evaluated by examining all of the SNPs within a 10 kB neighborhood surrounding the indicated causative genes. The smallest *p*-value obtained using the EMMA method for a SNP within this neighborhood serves as the “minimum *p*-value” for the causative gene. We also show the number of other SNPs in the mouse genome that had an equivalent or smaller *p*-value for each trait. The size of the genomic regions and the number of genes within 10 kB of these SNPs are shown, which provides an indication of the number of “false positive” correlations. The numbers of genes with codon changes are also shown. For the three binary response traits (AH response, Anthrax, and albinism), EMMA identified the causative gene. For the two quantitative traits tested, EMMA identified over 21,691 SNPs (corresponding to 66.1 MB encoding 922 genes) or 12,113 SNPs (64.7 MB, 1064 genes), with an equivalent or higher correlation than the known causative variants for survival after *C*. *albicans* infection (C5), or haloperidol-induced latency on day 30 (*Abcb5*), respectively. The causative genes for these quantitative traits had three or more distinct phenotypic responses, which indicates that methods that analyze only one SNP at a time are not optimal for analyzing traits where the causative genetic variants have more than two distinctive haplotypic groupings.
(DOCX)

S4 Table The top 10 genes (indicated by symbol) from the robustness analyses performed using the day 30, 60, and 120 latency data are shown. *Abcb5* (highlighted) ranked number 1, number 4, and number 3 on days 30, 60, and 120, respectively. Of note, if the small olfactory genes (Olfr392–398) and pseudogenes (Gm12329) were excluded, *Abcb5* would rank number 1 and number 2 on days 60 and 120, respectively.
(DOCX)

S5 Table Genes on Chromosome 12 whose genetic pattern was identified by HBCGM as having some level of correlation with the day 30 haloperidol-induced latency measurements for 17 inbred strains. The symbol, starting and ending position on Chromosome 12, and the calculated *p*-value and genetic effect size are shown for each gene. Three genes with SNPs that significantly altered an amino acid in the predicted protein sequence are indicated in orange, while two with minor amino acid substitutions are shown in yellow. *Abcb5* and Sp8 are collinear genes that had the highest correlation. Sp8 is a zinc finger transcription factor affecting limb development that is only expressed in liver and blood cells. Another gene of potential interest was Kcns3, which is a potassium voltage gated channel. However, Kcns3 is expressed in kidney, does not have any SNPs causing amino acid changes, and has a >10-fold lower level of correlation than does Abcb5.
(DOCX)

S1 Text Whole genome sequencing of inbred mouse strains facilitates genetic trait analysis. (DOCX)

## Abbreviations:

CNS: central nervous system
CSS: chromosome substitution strain
DESI-MSI: desorption electrospray ionization mass spectroscopic imaging
GWAS: genome wide association studies
HBCGM: haplotype-based computational genetic mapping
HE: hematoxylin and eosin
HIT: haloperidol-induced toxicity
MPTP: N-methyl-4-phenyl-1,2,3,6-tetrahydropyridine
NGS: next generation sequencing
SEM: standard error of the mean
