# Supplementary figures and images for: The Role of Abcb5 Alleles in Susceptibility to Haloperidol-Induced Toxicity in Mice and Humans
Source: PLoS Med. 2015 Feb 3;12(2):e1001782. doi: 10.1371/journal.pmed.1001782 (PMC4315575; doi:10.1371/journal.pmed.1001782)

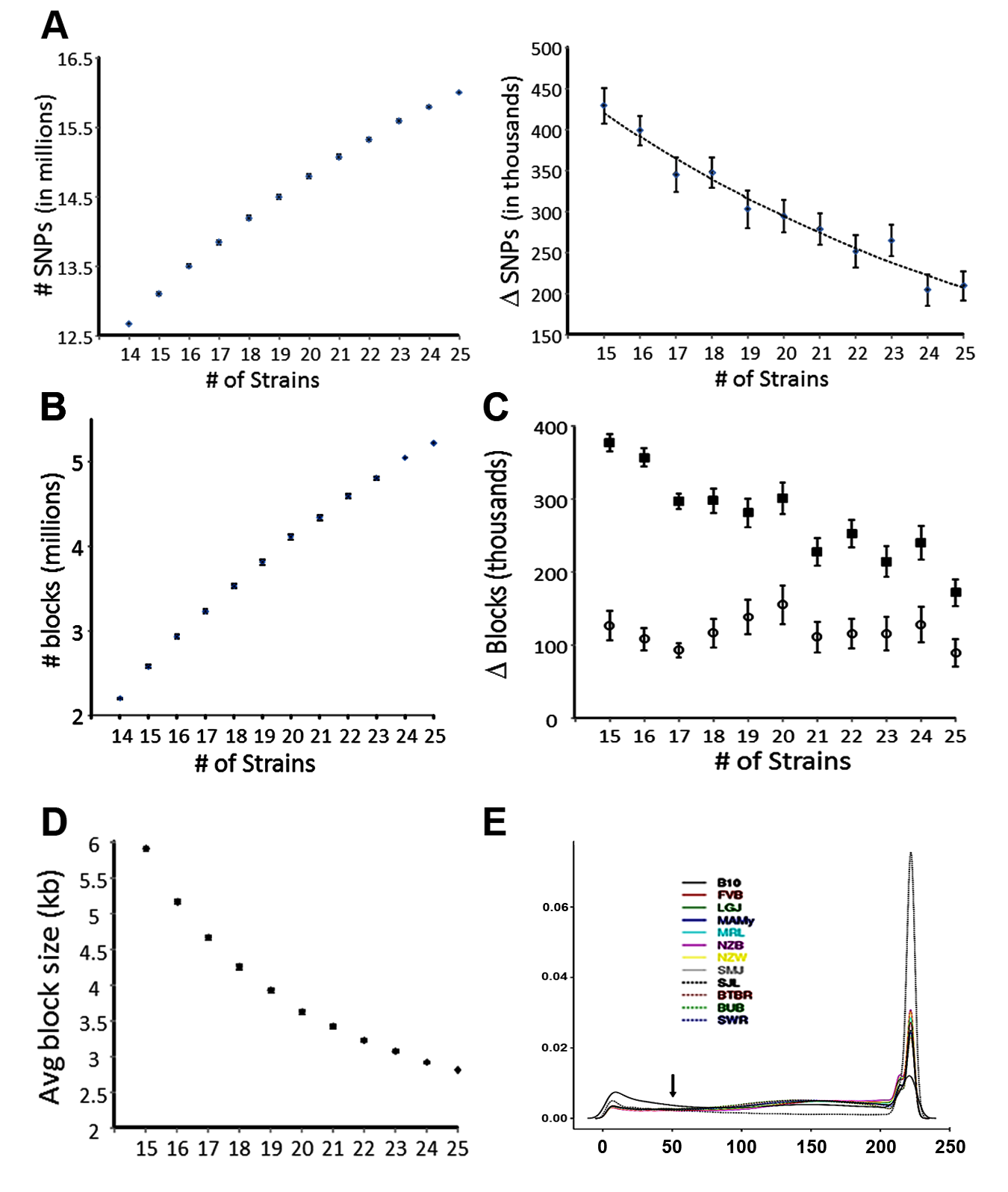

Supplement: S1 Fig — (A) The number of total (left panel) and added (right panel) SNPs identified after analysis of the genomic sequence obtained from the indicated number of additional strains. Beginning with the 12.6 M SNPs present in 14 strains, additional SNPs were identified by analyzing the sequence of 11 additional strains in a random order. This procedure was repeated 50 times, and the order of the added strains was varied. The left panel shows the total number of SNPs ± SEM from for each additional strain; while the right panel shows the number of added SNPs ± SEM for each added strain. Of note, the total number of SNPs increased significantly for each added strain. However, the number of new SNPs identified per additional strain analyzed decreased in an exponential fashion (blue line) as additional strains were evaluated. (B) The total number (± SEM) of haplotype blocks identified after inclusion of SNPs that were identified by analysis of the NGS sequence data obtained from the 15th through 25th strains that were analyzed is shown. These numbers were determined using the simulation described in (A). (C) The total numbers of additional haplotype blocks (± SEM) that were identified after incorporation of SNP data from the indicated number of additional analyzed strains are shown as diamonds. The numbers of added blocks (± SEM) that were produced as a result of new SNPs present in the additional analyzed strains are shown as circles. Of note, 30%–53% of new haplotype blocks are produced from newly identified SNPs, while the remainder are produced by new recombinations occurring in regions with previously known SNPs. (D) The average size of a haplotype block (± SEM) decreases as the additional allelic data are incorporated into the genetic map. (E) A density plot of the quality (QUAL) score for the SNP calls, which were determined by analysis of the sequence of each indicated strain using Samtools. The QUAL score reflects the likelihood that there is a genetic variant at each ide [file pmed.1001782.s003.tif]

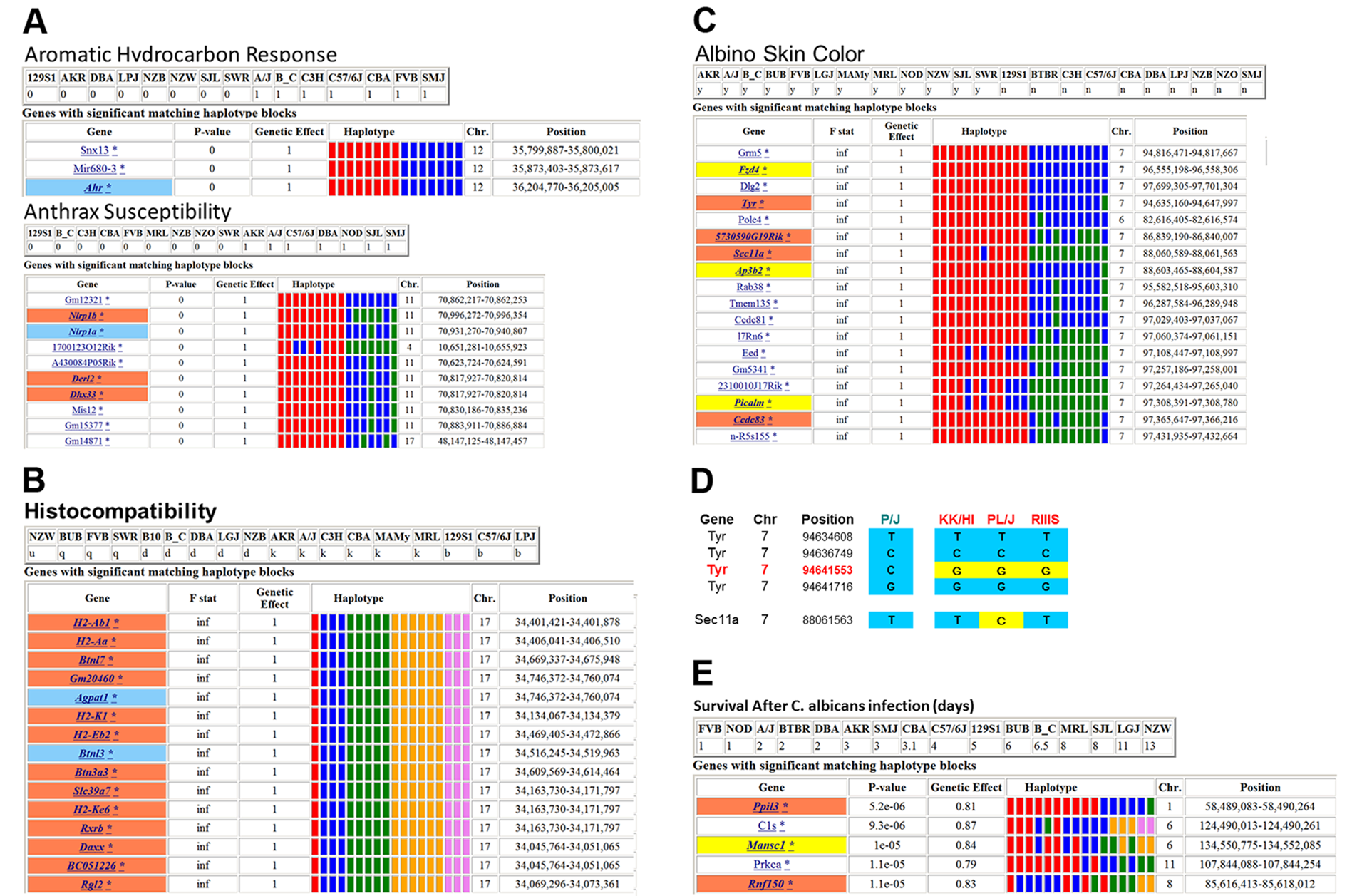

Supplement: S2 Fig — The p-value, and Genetic Effect Size were determined as previously described [8]. The genes within correlated haplotype blocks are indicated by their symbol; a blue, orange, yellow, or white background indicates whether SNPs altering a splice site, or causing a significant, minor, or no amino acid change within the predicted protein sequence are present, respectively. The haplotypic pattern is shown as colored rectangles arranged in the same order as the input data. Strains with the same colored rectangle have the same haplotype within the block at the indicated chromosome and position. The presence (y) or absence (n) of albinism in each strain is indicated in (C). (D) The association between SNP alleles in two candidate genes and albino status are shown for four additional strains: one strain (P/J) is non-albino (green) and three are albino (red). The alleles for each SNP are indicated by letter and by box color. Only the Cys103Ser alleles in the Tyr gene segregate with the albino status of these four strains. (TIF) [file pmed.1001782.s004.tif]

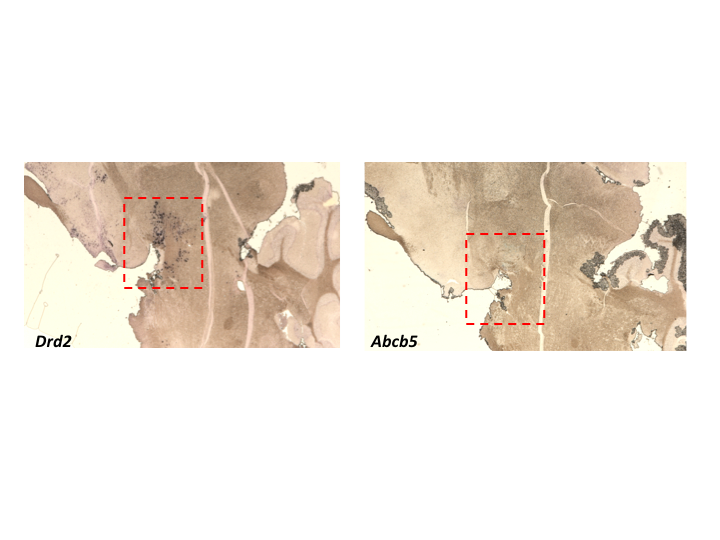

Supplement: S3 Fig — In situ hybridizations were performed using anti-sense probes for Abcb5 and Drd2. The dashed box region shows the substantia nigra region. The images of sagittal brain sections were obtained from a C57BL/6 mouse, and are shown at 1× magnification. (TIFF) [file pmed.1001782.s005.tiff]

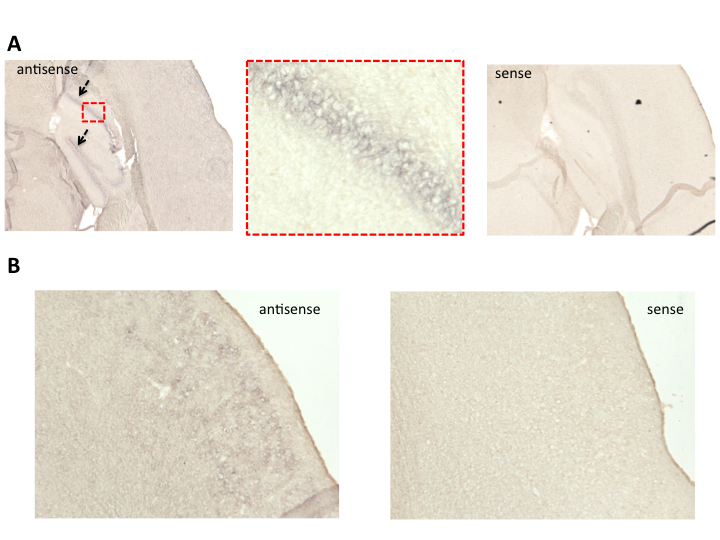

Supplement: S4 Fig — In situ hybridization was performed using anti-sense probes for Abcb5 on C57BL/6 mouse brain sections. As a negative-control, hybridizations were also performed using the sense probe on adjacent tissue sections. Abcb5 mRNA was expressed in a linear pattern within the hippocampus (arrows). In addition, Abcb5 was also faintly expressed in the frontal cortex. The images are shown at 4× magnification, and a magnified view (20×) of the boxed region in (A) is shown in the center image. (TIFF) [file pmed.1001782.s006.tiff]

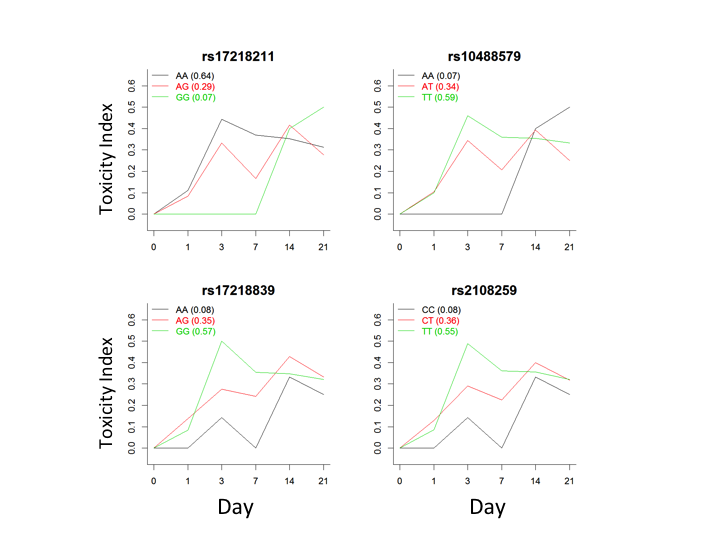

Supplement: S5 Fig — The average combined toxicity measurement was calculated for each genotype at each SNP, and then plotted according to the colors shown in the legend. (TIFF) [file pmed.1001782.s007.tiff]
