# Supplementary material for: The Role of Abcb5 Alleles in Susceptibility to Haloperidol-Induced Toxicity in Mice and Humans
Source: PLoS Med. 2015 Feb 3;12(2):e1001782. doi: 10.1371/journal.pmed.1001782 (PMC4315575; doi:10.1371/journal.pmed.1001782)
Supplement: S1 Table — The amount of genomic sequence (Gb), fold coverage, numbers of SNPs, or indels relative to the C57BL/6 reference sequence (or uniquely present in each strain) are shown. The sequence data for the first 12 strains was obtained at Stanford, C57BL6 is the reference sequence, and Keane and colleagues [1] produced the sequence data for the other 13 strains. The sequence coverage and SNP variants identified by analysis of NGS data for 26 mouse genomes. The amount of genomic sequence (Gb), fold coverage, numbers of SNPs, or indels relative to the C57BL/6 reference sequence (or uniquely present in each strain) are shown. (DOCX) [file pmed.1001782.s008.docx]

**Table S1**. The sequence coverage and SNP variants identified by analysis of NGS data for 26 mouse genomes. The amount of genomic sequence (Gb), fold coverage, numbers of SNPs or indels relative to the C57BL/6 reference sequence (or uniquely present in each strain) are shown. The sequence data for the first 12 strains was obtained at Stanford, C57BL6 is the reference sequence, and Keane et al [[1](#_ENREF_1)] produced the sequence data for the other 13 strains.

| Strain | Gb | Fold  Coverage | Number of SNPs | Number of Unique  SNPs | Number of Indels | Number of Unique  Indels |
| --- | --- | --- | --- | --- | --- | --- |
| B10.D2 n2SnJ | 73.2 | 24 | 316,899 | 29,940 | 42,738 | 5,977 |
| BTBR | 59 | 20 | 3,494,456 | 86,000 | 410,588 | 25,176 |
| BUB | 59.2 | 20 | 4,052,634 | 180,499 | 481,897 | 39,072 |
| FVB | 83 | 28 | 4,035,843 | 86,674 | 501,131 | 25,337 |
| LGJ | 73.4 | 24 | 4,189,165 | 103,869 | 490,272 | 27,073 |
| MA/My | 78 | 26 | 3,825,893 | 290,231 | 448,568 | 46,668 |
| MRL/MpJ | 93.2 | 31 | 4,255,154 | 19,969 | 517,633 | 17,773 |
| NZB/BlnJ | 94.2 | 31 | 4,674,253 | 275,931 | 591,120 | 66,754 |
| NZW/LacJ | 90.6 | 30 | 4,816,980 | 355,296 | 584,522 | 64,989 |
| SJL | 165 | 55 | 5,086,164 | 414,597 | 703,374 | 46,354 |
| SMJ | 83 | 28 | 4,448,252 | 458,620 | 529,108 | 75,978 |
| SWR | 59.9 | 20 | 4,201,015 | 191,783 | 507,174 | 42,808 |
| 129P2/OlaHsd | 115.5 | 43.8 | 4511910 | 12643 | 710137 | 46841 |
| 129S1/SvImJ | 71.9 | 27 | 4566578 | 32049 | 531052 | 30625 |
| 129S5SvEvBrd | 50.3 | 19.1 | 4235649 | 12139 | 418552 | 13572 |
| A/J | 70.4 | 24.7 | 4226537 | 65232 | 459829 | 22367 |
| AKR/J | 107.2 | 40.6 | 4538389 | 167278 | 592213 | 57011 |
| Balb/cJ | 65.7 | 24.9 | 3954750 | 63043 | 432082 | 18772 |
| C3H/HeJ | 92.8 | 35.2 | 4550468 | 83251 | 553139 | 35682 |
| C57BL/6J |  |  | REF | 8454 | REF | 2327 |
| C57BL/6NJ | 77.3 | 29 | 11077 | 1901 | 4426 | 526 |
| CBA/J | 77.4 | 29.3 | 4477789 | 61166 | 462785 | 18396 |
| DBA/2J | 65.1 | 24.7 | 4530098 | 226229 | 494595 | 39633 |
| LP/J | 73 | 27.7 | 4644304 | 106081 | 468762 | 23295 |
| NOD/LtJ | 75.9 | 28.8 | 4462384 | 224066 | 481322 | 43041 |
| NZO/HlLtJ | 45.7 | 17.3 | 4642787 | 181857 | 507793 | 35979 |
