# Supplementary material for: The Role of Abcb5 Alleles in Susceptibility to Haloperidol-Induced Toxicity in Mice and Humans
Source: PLoS Med. 2015 Feb 3;12(2):e1001782. doi: 10.1371/journal.pmed.1001782 (PMC4315575; doi:10.1371/journal.pmed.1001782)
Supplement: S2 Table — The aryl hydrocarbon response (AHR), major histocompatibility complex (MHC) H2 haplotypes (MHC H2), litter size (MPD: 31408), and macrophage susceptibility to the Bacillus anthracis lethal toxin (Anthrax MPD: 1501) were obtained from [8] and from the Mouse Phenome Database (http://phenome.jax.org/). Since the MHC H2 haplotypes for NOD/ShiLtJ (g7), SJL/J (s), and SM/J (v) are unique and each is distinct from that of the other 18 strains; they were not used for the HBCGM results shown in this paper. We can include no more than five different phenotypes in a mapping experiment since haplotype blocks with up to five haplotypes are analyzed. The albino status (Albino) was obtained from the Jackson Laboratory description of mouse strains (http://www.jax.org/). The Candida albicans (C. albicans) survival data was obtained from [11]. (B) The phenotypic dataset used for analysis of haloperidol-induced latency. The measured haloperidol-induced latencies (seconds) on days 0 (MPD: 39407), 3 (MPD: 39408), 7 (MPD: 39409), 30 (MPD: 39410), 60 (MPD: 39411), and 120 (MPD: 39445); and the plasma haloperidol levels on day 30 (MPD: 39403) were obtained from the Mouse Phenome Database (MPD) (http://phenome.jax.org). The number of mice examined, average measurement, and the standard deviation are shown for each of the 16 indicated strains. (DOCX) [file pmed.1001782.s009.docx]

**Table S2A**. The phenotypic data used for HBCGM of 5 traits. The aryl hydrocarbon response (**AHR),** MHC H2 haplotypes (MHC H2), litter size (MPD: 31408), and macrophage susceptibility to the *Bacillus anthracis* lethal toxin (Anthrax MPD: 1501) were obtained from [[8](#_ENREF_8)] and from the Mouse Phenome Database (<http://phenome.jax.org/>). Since the MHC H2 haplotypes for NOD/ShiLtJ (g7), SJL/J (s) and SM/J (v) are unique and each is distinct from that of the other 18 strains; they were not used for the HBCGM results shown in this paper. We can include no more than 5 different phenotypes in a mapping experiment since haplotype blocks with up to 5 haplotypes are analyzed. The albino status (Albino) was obtained from the Jackson Laboratory description of mouse strains (<http://www.jax.org/>). The *Candida albicans* (*C. albicans*) survival data was obtained from [[11](#_ENREF_11)].

| Strain | AHR | MHC H2 | Anthrax | Albino | *C. albicans* |
| --- | --- | --- | --- | --- | --- |
| 129S1/SvImJ | 0 | B | 0 | n | 5 |
| A/J | 1 | K | 1 | y | 2 |
| AKR/J | 0 | K | 1 | y | 3 |
| B10.D2-H2/n2SnJ |  | D |  |  |  |
| Balb/cJ | 1 | D | 0 | y | 6.5 |
| BTBR T+ Itpr3tf/J |  |  |  | n | 2 |
| BUB/BnJ |  | Q |  | y | 6 |
| C3H/HeJ | 1 | K | 0 | n |  |
| C57BL/6J | 1 | B | 1 | n | 4 |
| CBA/J | 1 | K | 0 | n | 3.1 |
| DBA/2J | 0 | D | 1 | n | 2 |
| FVB/NJ | 1 | Q | 0 | y | 1 |
| LG/J |  | D |  | y | 11 |
| LP/J | 0 | B |  | n | 5.5 |
| MA/MyJ |  | K |  | y |  |
| MRL/MpJ |  | K | 0 | y | 8 |
| NOD/ShiLtJ |  |  | 1 | y | 1 |
| NZB/BlnJ | 0 | D | 0 | n |  |
| NZO/HlLtJ |  |  | 0 | n |  |
| NZW/LacJ | 0 | U |  | y | 13 |
| SJL/J | 0 |  | 1 | y | 8 |
| SM/J | 1 |  | 1 | n | 3 |
| SWR/J | 0 | Q | 0 | y |  |

**Table S2B.** The phenotypic dataset used for analysis of haloperidol-induced latency. The measured haloperidol-induced latencies (seconds) on days 0 (MPD: 39407) 3 (MPD: 39408), 7 (MPD: 39409), 30 (MPD: 39410), 60 (MPD: 39411) and 120 (MPD: 39445); and the plasma haloperidol levels on day 30 (MPD: 39403) were obtained from the Mouse Phenome Database (**MPD**) (<http://phenome.jax.org>). The number of mice examined, average measurement and the standard deviation are shown for each of the 16 indicated strains.
