# Supplementary material for: The Role of Abcb5 Alleles in Susceptibility to Haloperidol-Induced Toxicity in Mice and Humans
Source: PLoS Med. 2015 Feb 3;12(2):e1001782. doi: 10.1371/journal.pmed.1001782 (PMC4315575; doi:10.1371/journal.pmed.1001782)
Supplement: S3 Table — Genetic variation within the indicated causative genes was among the most highly correlated candidates identified by HBCGM. After EMMA analyzed the same phenotypic data, its results were evaluated by examining all of the SNPs within a 10 kB neighborhood surrounding the indicated causative genes. The smallest p-value obtained using the EMMA method for a SNP within this neighborhood serves as the “minimum p-value” for the causative gene. We also show the number of other SNPs in the mouse genome that had an equivalent or smaller p-value for each trait. The size of the genomic regions and the number of genes within 10 kB of these SNPs are shown, which provides an indication of the number of “false positive” correlations. The numbers of genes with codon changes are also shown. For the three binary response traits (AH response, Anthrax, and albinism), EMMA identified the causative gene. For the two quantitative traits tested, EMMA identified over 21,691 SNPs (corresponding to 66.1 MB encoding 922 genes) or 12,113 SNPs (64.7 MB, 1064 genes), with an equivalent or higher correlation than the known causative variants for survival after C. albicans infection (C5), or haloperidol-induced latency on day 30 (Abcb5), respectively. The causative genes for these quantitative traits had three or more distinct phenotypic responses, which indicates that methods that analyze only one SNP at a time are not optimal for analyzing traits where the causative genetic variants have more than two distinctive haplotypic groupings. (DOCX) [file pmed.1001782.s010.docx]

**Table S3.** The results obtained using the EMMA method [[5](#_ENREF_5)] for evaluating the phenotypic data for 5 traits. Genetic variation within the indicated causative genes was among the most highly correlated candidates identified by HBCGM. After EMMA analyzed the same phenotypic data, its results were evaluated by examining all of the SNPs within a 10 KB neighborhood surrounding the indicated causative genes. The smallest p value obtained using the EMMA method for a SNP within this neighborhood serves as the ‘minimum p value’ for the causative gene. We also show the number of other SNPs in the mouse genome that had an equivalent or smaller p value for each trait. The size of the genomic regions and the number of genes within 10 kB of these SNPs are shown, which provides an indication of the number of ‘false positive’ correlations. The numbers of genes with codon changes are also shown. For the three binary response traits (AH response, Anthrax, and albinism), EMMA identified the causative gene. For the 2 quantitative traits tested, EMMA identified over 21,691 SNPs (corresponding to 66.1 MB encoding 922 genes) or 12,113 SNPs (64.7 MB, 1064 genes), with an equivalent or higher correlation than the known causative variants for survival after *C. albicans* infection (*C5*), or haloperidol-induced latency on day 30 (*Abcb5*), respectively. The causative genes for these quantitative traits had 3 or more distinct phenotypic responses, which indicates that methods that analyze only one SNP at a time are not optimal for analyzing traits where the causative genetic variants have more than two distinctive haplotypic groupings.
