# Supplementary material for: The Role of Abcb5 Alleles in Susceptibility to Haloperidol-Induced Toxicity in Mice and Humans
Source: PLoS Med. 2015 Feb 3;12(2):e1001782. doi: 10.1371/journal.pmed.1001782 (PMC4315575; doi:10.1371/journal.pmed.1001782)
Supplement: S4 Table — Abcb5 (highlighted) ranked number 1, number 4, and number 3 on days 30, 60, and 120, respectively. Of note, if the small olfactory genes (Olfr392–398) and pseudogenes (Gm12329) were excluded, Abcb5 would rank number 1 and number 2 on days 60 and 120, respectively. (DOCX) [file pmed.1001782.s011.docx]

**Table S4**. The top 10 genes (indicated by symbol) from the robustness analyses performed using the day 30, 60 and 120 latency data are shown. *Abcb5* (highlighted) ranked #1, #4 and #3 on days 30, 60 and 120, respectively. Of note, if the small olfactory genes (Olfr392-398) and pseudogenes (Gm12329) were excluded, *Abcb5* would rank #1 and #2 on days 60 and 120, respectively.

| **Day 30** | **Score** | **Day 60** | **Score** | **Day 120** | **Score** |
| --- | --- | --- | --- | --- | --- |
| Abcb5 | 872.20 | Olfr393 | 1097.9 | Olfr393 | 846.27 |
| Sp8 | 871.84 | Olfr392 | 1091.1 | Macc1 | 845.03 |
| Wnt2b | 863.89 | Gm12329 | 1078.3 | Abcb5 | 844.05 |
| Csmd1 | 862.52 | Abcb5 | 1072.3 | Gm12327 | 842.85 |
| Olfr392 | 859.76 | Olfr397 | 1071 | Olfr392 | 841.57 |
| Olfr393 | 858.25 | Olfr398 | 1071 | Gm12329 | 841.5 |
| Olfr397 | 854.54 | Olfr394 | 1069.7 | Vstm2l | 840.94 |
| Ctnna2 | 853.84 | Gm12984 | 1069.3 | Olfr398 | 840.79 |
| Epha6 | 853.12 | Rap1gap2 | 1068.7 | Olfr397 | 840.79 |
| Olfr398 | 851.79 | Olfr390 | 1068.7 | Olfr394 | 840.60 |
