# Supplementary material for: The Role of Abcb5 Alleles in Susceptibility to Haloperidol-Induced Toxicity in Mice and Humans
Source: PLoS Med. 2015 Feb 3;12(2):e1001782. doi: 10.1371/journal.pmed.1001782 (PMC4315575; doi:10.1371/journal.pmed.1001782)
Supplement: S5 Table — The symbol, starting and ending position on Chromosome 12, and the calculated p-value and genetic effect size are shown for each gene. Three genes with SNPs that significantly altered an amino acid in the predicted protein sequence are indicated in orange, while two with minor amino acid substitutions are shown in yellow. Abcb5 and Sp8 are collinear genes that had the highest correlation. Sp8 is a zinc finger transcription factor affecting limb development that is only expressed in liver and blood cells. Another gene of potential interest was Kcns3, which is a potassium voltage gated channel. However, Kcns3 is expressed in kidney, does not have any SNPs causing amino acid changes, and has a >10-fold lower level of correlation than does Abcb5. (DOCX) [file pmed.1001782.s012.docx]

**Table S5**. Genes on chromosome 12 whose genetic pattern was identified by HBCGM as having some level of correlation with the day 30 haloperidol-induced latency measurements for 17 inbred strains. The symbol, starting and ending position on chromosome 12, and the calculated p-value and genetic effect size are shown for each gene. Three genes with SNPs that significantly altered an amino acid in the predicted protein sequence are indicated in orange, while two with minor amino acid substitutions are shown in yellow. *Abcb5* and *Sp8* are collinear genes that had the highest correlation. Sp8 is a zinc finger transcription factor affecting limb development that is only expressed in liver and blood cells. Another gene of potential interest was *Kcns3,* which is a potassium voltage gated channel. However, *Kcns3* is expressed in kidney, does not have any SNPs causing amino acid changes, and has a >10-fold lower level of correlation than does *Abcb5*.
