# Supplementary material for: The Role of Abcb5 Alleles in Susceptibility to Haloperidol-Induced Toxicity in Mice and Humans
Source: PLoS Med. 2015 Feb 3;12(2):e1001782. doi: 10.1371/journal.pmed.1001782 (PMC4315575; doi:10.1371/journal.pmed.1001782)
Supplement: S1 Text — (DOCX) [file pmed.1001782.s013.docx]

**Supplemental Information**

**Whole Genome Sequencing of Inbred Mouse Strains Facilitates Genetic Trait Analysis**

Ming Zheng^1#^, Haili Zhang^1#^, David L. Dill^2^, J. David Clark^3^, Susan Tu^2^, Arielle L. Yablonovitch^4^, Meng How Tan^4^, Rui Zhang^4^, Dan Rujescu^5^, Manhong Wu^1^, Lino Tessarollo^6^, Wilfred Vieira^7^, Michael M Gottesman^7^, Suhua Deng^8^, Livia S. Eberlin^9^, Richard N. Zare^9^, Jean-Martin Billard^10^, Jean-Pierre Gillet^10^, Jin Billy Li^4*^ and Gary Peltz^1*^

**A comprehensive genetic map for 26 inbred strains**

The SNP database used for the genetic mapping experiments described here has 16.0 million SNPs with alleles covering 26 strains, including the 12 strains sequenced here, 13 strains of the 17 strains analyzed in [[1](#_ENREF_1)], and the reference C57BL/6 strain. Since we previously found that inclusion of SNP data from wild-derived strains (CAST/Ei, PWK/Ph, SPRET/Ei and WSB/Ei) disrupts the haplotypic patterns present in the classical inbred strains [[2](#_ENREF_2)], the SNP alleles in these strains from the data in [[1](#_ENREF_1)] were not included. We sequenced the genome of 12 additional inbred strains because the power of HBCGM to identify a genetic locus is increases as the number of strains is increased [[3](#_ENREF_3)]. The extent of sequence coverage and the genomic variants discovered in these 26 strains are summarized in **Table S1**. Genomic variants are defined as sequences that are different from the C57BL/6J reference strain (MGSCv37 assembly); and 3.5 to 5 million SNPs were identified in each of the analyzed strains, except for B10.D2, which is genetically close to the reference strain. The fold coverage for 11 of the 12 strains sequenced here are comparable (between 20 to 31-fold) to those (17 to 44-fold) in Keane et al [[1](#_ENREF_1)]. The only exception is SJL/J (the first strain that we sequenced), which had a higher (55-fold) level of sequence coverage. Despite the increased sequence coverage, the percentage of high confidence variant calls in SJL/J was similar to that in the other strains, but the advantage of the higher sequence coverage becomes evident when variant calls with a very high confidence level are considered (**Figure S1E**). Although the total number of SNPs found in each analyzed strain was similar, there was a large difference in the number of SNPs with alleles that were uniquely present in a single strain. For example, SM/J has >458K unique SNPs, while MRL (<20K) and LG/J (<105K) had substantially smaller numbers of unique SNPs. This indicates that there are large differences in the number of spontaneous mutations that occurred after the strains diverged.

The ~12 million SNPs identified in the 14 initially analyzed strains increased to 16 million SNPs after the 12 additional strains were analyzed (**Fig. S1**). The number of newly identified SNPs increased by an average of ~429,000 after the first additional (i.e. 15^th^) strain was analyzed, but decreased in an approximately exponential fashion to ~209,000 new SNPs per strain after analysis 11^th^ additional strain (Fig. S1). However, according to our projections, the number of new SNPs per added strain decreases to ~70,000 only after 40 strains are analyzed. Since haplotype blocks are used in our genetic mapping method, we examined the impact that incorporation of SNP data from additional strains had upon the haplotype map. The number of haplotype blocks increased and the average size of a haplotype block decreased after the allelic data from additional strains were incorporated (**Fig. S1**). The increased number of haplotype blocks could result from the introduction of new SNPs or from new recombinations occurring within regions containing known polymorphisms. We found that 30-53% of the new haplotype blocks formed after incorporation of allelic data from additional strains resulted from newly discovered SNPs (Fig. S1B).

**Comments on the SNP database and HBCGM method**

(i) The use of whole genome sequence data substantially increases the resolution of the haplotype map to sub-genic granularity, and improves association-mapping results. While the size of a haplotype block varies across the mouse genome, the average size of a haplotype block is below 5 kB (Fig. S1B), which is less than the average size of a mouse gene (~30 kB). Since high-density whole-genome SNP data contains almost all of the variations present in a mouse genome, the use of whole genome sequence ensures that a true causative locus is directly analyzed by HBCGM. As reviewed in [[4](#_ENREF_4)], genetic analyses that includes a causative SNP produces results that are superior to those that only analyze tagging SNPs. Also, for a phenotypic response that uniquely appears in one inbred strain, the significant number of newly identified SNPs obtained from whole genome sequencing, ensures that the causative genetic factor can be identified.

(ii) Our current HBCGM method does not correct for the population structure that exists among the inbred strains, which could cause an increase in the number of false positive (FP) associations [[5](#_ENREF_5)]. However, correcting for population structure inevitably increases the chance for producing false negative (FN) results. In practice, FN results are much more serious type of error than are FP results: if an association method misses a true causative genetic variant, it is forever removed from consideration. Although a correction for the population structure can be readily performed in HBCGM using the mixed effect model [[5](#_ENREF_5)], we strongly favor using methods that do not increased the of FN results. Therefore, our current practice is not to correct for population structure, and to assume that many correlations are false positives. We use additional (orthogonal) information (e.g. gene expression, metabolomics data, annotation information) to filter out FP results after the analysis is computationally performed, and to assist in identifying the likely true positive using methods described elsewhere [[6](#_ENREF_6)]. Although there is certainly room for different approaches, we have a substantial track record of successfully identifying genetic factors affecting a large number of biomedical traits [[4](#_ENREF_4)] with this method, which indicates it has some validity.

(iii) The p values are not corrected for multiple testing. From a mathematical perspective, due to the monotonicity of the process used for p value correction, the traditional (e.g. Bonferroni’s or Benjamini-Hochberg [[7](#_ENREF_7)]) methods used for multiple-testing correction are essentially equivalent to setting a cutoff for the raw p values that correspond to the cutoff set for corrected p values,. Because of this equivalence, the most important step is to set an appropriate cutoff, which is designed to filter out the FP findings, while controlling the FN rate. Since ~ 5 million haplotype blocks are tested, application of a multiple-testing correction will be too conservative, and will produce an extremely high FN rate. Since a true causative gene that is removed from consideration as a FN can’t be recovered, a FN has much more serious consequences than maintaining a number of FP results, which can be subsequently filtered using orthogonal information [[6](#_ENREF_6)]. Therefore, in practice, we find that applying a flexible cutoff for evaluating the raw p values improves the probability of identifying a true causative gene, and we then use additional criteria to further filter through the correlated genes.

(iv) Wild-derived strains (CAST/Ei, SPRET/Ei) are not routinely included in HBCGM studies. As discussed elsewhere [[2](#_ENREF_2)], their extreme genetic divergence from the laboratory strains produces a genetic map with a larger number of SNPs, but their inclusion actually prevents association studies involving classical inbred from identifying causative genetic factors. As shown for analysis of chromosome 7 SNP data [[2](#_ENREF_2)], inclusion of wild-derived strains produces a genetic map where 25 to 50% of the genome has haplotype blocks where the wild-derived strain has a unique haplotype. In other words, if a wild-derived strain has a unique phenotype, an association study will identify candidates in up to half of the genome.

(v) HBCGM can only be used to evaluate mono- (or oligo-) genic traits where the causative locus has a genetic effect size above a minimum threshold, which is dependent upon the number of strains analyzed [[2](#_ENREF_2)]. It cannot be used to evaluate complex traits at present. Many previous analyses, which only analyzed the phenotypic responses in a small number of inbred strains, have underestimated the genetic effect size for genetic factors affecting a number of important biomedical traits [[4](#_ENREF_4)]. If analysis of a larger number of strains reveals a wider range of phenotypic responses among the inbred stains, this increases the genetic effect size, which often enables HBCGM to be used to identify causative genetic factors.

**Analysis of 4 phenotypic traits using HBCGM**

To determine if whole genome data could be productively utilized for genetic studies, we analyzed the available phenotypic data for 4 traits where the genetic factors responsible for inter-strain differences were already known (**Table S2**). For example, genetic variation within the *aromatic hydrocarbon receptor* (*Ahr*) gene is known to be responsible for the differential response of the inbred strains differ to aromatic hydrocarbon exposure [[8](#_ENREF_8)]. HBCGM analysis of aromatic hydrocarbon response data for 15 strains (**Table S2**) indicated that *Ahr* was one of the top 3 gene candidates identified by the mapping software (**Fig. S2A**). The other two correlated genes *(Snx13* and *Mir680-3*) could be easily eliminated by inspection of available annotation information, since they are not even protein coding genes. We next examined the susceptibility of murine macrophages to apoptosis induced by the *Bacillus anthracis* lethal toxin, because susceptibility differences have been shown to be caused by genetic variation within the *Nalp1b* gene [[9](#_ENREF_9)]. The co-linear *Nalp1a and Nalp1b* genes were 2 of only 4 genes identified by analysis of the available response data for 16 strains (Fig. S2A). We next analyzed the *K* locus phenotypes of the Major Histocompatibility Complex (*MHC*), which is a qualitative trait with at least 5 different response types observed among the 18 analyzed strains (Table S2). The categorical response types (indicated by letters: *b, d, k, q, u*) were transformed into points in multi-dimensional metric space, such that each K locus category was equidistant from any other category. The mapping software identified contiguous haplotype blocks clustered within a 0.8 MB region on chromosome 17, which contains the known MHC locus (**Fig. S2B**).

The genetic basis for the albino skin type that appears in some inbred strains is determined by the alleles at a Cys103Ser SNP within the tyrosinase (*Tyr*) gene [[10](#_ENREF_10)]. HBCGM of the albino status data for 22 inbred strains identified only 4 candidate genes, which had a significant codon change and at most 3 haplotypes in the matching perfect block. Of note, these genes were in a contiguous region on chromosome 7; and only two (*Tyr* and *Sec11a*) were expressed in skin (**Fig. S2C**). To investigate whether analysis of additional strains could specifically pinpoint the known causative factor, the association between SNPs alleles within the 2 gene candidates that were expressed in skin and albino status was examined in 4 additional strains whose albino status was characterized (Table S1). Of note, only Cys103Ser SNP alleles within the *Tyr* gene segregated with the albino status of these 4 strains (**Fig. S2D**). It was of interest to determine why phenotypic data from 26 strains was required for the specific identification of the causative gene. Therefore, we examined the pattern of genetic variation within the 13 kb haplotype block within the *Tyr* gene, which contained 33 SNPs. SM/J is a non-albino strain with unique alleles at 31 of these SNPs; it shares alleles at 2 SNPs with the albino strains, and the alleles at 2 other SNPs are shared with the non-albino strains. Thus, the 33 SNPs in the *Tyr* gene could distinguish all of the albino strains from the non-albino strains, with the only exception being that SM/J has it own haplotypic pattern. Even though one of the non-albino strains had a very distinct haplotype, as the follow-up genetic association study demonstrates, the causative gene could be identified using whole genome sequence information covering a few additional strains.

To investigate whether HBCGM could analyze quantitative trait data, we evaluated the median survival data after *Candida albicans* infection (range: 1-13 days) [[11](#_ENREF_11)] (Table S2) that was available for 16 of the 26 fully sequenced strains. We recently found that allelic variation within complement factor 1s (*C1s*) affected survival after fungal infection, and that a combinatorial genetic model, involving an interaction between complement factor 5 (*Hc* or *C5*) and *C1s* alleles*,* could accurately predict inbred strain survival after infection [[11](#_ENREF_11)]. HBCGM results indicated that genetic variation within *C1s* had the 2^nd^ strongest correlation with survival (Figure S2C). Of note, the pattern of genetic variation within *C5* also correlated with survival, (p value = 0.00016), bit it had a lower degree of correlation than *C1s* (ranked #228).

**Comparison of Genetic Mapping Methods**

We also examined the results obtained using another genetic analysis method [the efficient mixed-model association (or **EMMA**) method [[5](#_ENREF_5)]], which analyzes the correlation between phenotypic data and alleles at a single SNP. EMMA identified the known causative gene for the 3 binary response traits (aromatic hydrocarbon response, anthrax, and albinism). For these binary phenotypes, which are entirely determined by the alleles at a single SNP, analyses based upon individual SNPs or haplotypes have the same detection power. However, EMMA could not analyze the qualitative trait (MHC response) with 5 different response categories; nor could it identify the causative gene for either of the quantitative traits tested (**Table S4)**. The causative genes for the traits that could not be analyzed by EMMA had 3 or more distinct phenotypic responses. Thus, methods that analyze one SNP at a time are not optimal for analyzing traits where the causative genetic variants have more than two haplotypic groupings.

The organization of genetic information into haplotypic patterns appears to improve genetic mapping results, especially for analysis of traits with three or more distinct types of responses. By organizing individual SNPs into haplotype blocks, genetic patterns with 3 or more strain groupings can be created, which can be correlated with the more complex phenotypic response patterns that are observed when larger numbers of inbred strains are examined. Since the phenotypic effect of many genetic variants are known to be ‘context’ dependent [[12](#_ENREF_12)], haplotype-block based analyses may be better able to incorporate some of the background effect than do analyses of single SNPs.

***Analysis of HIT data***. All strains exhibited a minimal pre-treatment latency (0 to 4 seconds), but there was a large inter-strain variation in the latency after 30 days of haloperidol treatment (0 to 234 seconds) (**Table S2B**). The prolonged latency on day 30 was a haloperidol-induced effect since: there was no correlation between the day 0 and day 30 latencies (adjusted p-value for Spearman’s *rho* = 0.64); and the latencies increased after 3, 7, 60, and 120 days of haloperidol treatment, and the relative magnitude of the strain response was maintained across all treatment days (adjusted p-values for Spearman’s *rho* were 6.3x10^-6^, 6.3x10^-6^, 1.3x10^-8^, 1.2x10^-5^). The inter-strain latency differences were not due to different rates of haloperidol metabolism, since the day 30 latency and plasma haloperidol levels were not correlated (adjusted p-value for Spearman’s *rho* = 0.64). This was expected since the effect of CNS-acting drugs is dependent upon the brain (and not the plasma) drug concentration, and brain haloperidol levels can be 20-fold higher than those in plasma.

**Supplemental Methods**

*Expanded association study for albinism*. The SNP data for 9 genes was obtained from published SNP datasets [[13](#_ENREF_13)].

*Determination Abcb5 haplotypes*. PCR amplification and sequencing of the amplicons was performed as described above. The 6 primer sets used for amplification of 281, 248, 244, 297, 300, and 357 base pair regions within Abcb5 are shown below.

| Gene | Chromosome | Sites | Forward Primers | Reverse Primers |
| --- | --- | --- | --- | --- |
| Abcb5 | 12 | 120106472 | AAAGTATGTATCTCCATTTCTCAGGA | TCCCAAAGTCAGGTAGTAAAACAA |
| Abcb5 | 12 | 120156610 | CCCCCACCTTGTAGTCTTTG | TGTGGTAGCACACAGGCTCT |
| Abcb5 | 12 | 120165757  120165763 | GCGGAGCCCTCAGGTACT | ATGACATCCGAGCCCAAA |
| Abcb5 | 12 | 120179019 | GGCCAATAGAAAACCCAGAT | TTCCTTCTGGGTCATAACTGC |
| Abcb5 | 12 | 120190973 | TGGCAGACACTATGATGAATCC | GGTATTTCTCTGGCACACTTTG |
| Abcb5 | 12 | 120203759  120203769 | GGAACCAATGTGGAAGCAAT | CAGCCGCTTCTGTGGTAGAC |

*EMMA analysis*. The phenotypic datasets were formatted according to the EMMA-specific guidelines, and the data was analyzed using the EMMA server (<http://mouse.cs.ucla.edu/emmaserver/>) with the 4-million candidate SNP set as of March 28, 2013. The results were downloaded and analyzed as follows: 1) all SNPs within a 10 KB neighborhood of the causative gene were identified and the smallest p-value for SNPs in this region was determined; 2) All SNPs with a p value that was less than or equal to the value determined in step 1 were identified; 3) The size of the genomic regions covered by the SNPs identified in step 2, which was defined as the non-redundant regions within 10 KB of any of these SNPs, was determined. 4) The SNPs identified in step 2 were annotated using predictive gene models from Ensembl version 65. The number of candidate genes identified by EMMA (defined as those genes that are within a 10KB neighborhood of a SNP identified in step 2) as well as candidate genes with codon-changing SNPs was identified using the annotation information.

**Supplemental References**

1. Keane TM, Goodstadt L, Danecek P, White MA, Wong K, et al. (2011) Mouse genomic variation and its effect on phenotypes and gene regulation. Nature 477: 289-294.

2. Wang J, Peltz G (2005) Haplotype-Based Computational Genetic Analysis in Mice. Computational Genetics and Genomics: New Tools for Understanding Disease. Totowa, New Jersey: Humana Press Inc. pp. 51-70.

3. Wang J, Liao G, Usuka J, Peltz G (2005) Computational Genetics: From Mouse to Man? Trends in Genetics 21: 526-532.

4. Zheng M, Dill D, Peltz G (2012) A better prognosis for genetic association studies in mice. Trends Genet 28: 62-69.

5. Kang HM, Zaitlen NA, Wade CM, Kirby A, Heckerman D, et al. (2008) Efficient control of population structure in model organism association mapping. Genetics 178: 1709-1723.

6. Zheng M, Shafer SS, Liao G, Liu H-H, Peltz G (2009) Computational Genetic Mapping in Mice: ‘The Ship has Sailed’. Science Translational Medicine 1: 3ps4.

7. Benjamini Y, Hochberg Y (1995) Controlling the false discovery rate: a practical and powerful approach to multiple testing Journal of the Royal Statistical Society Series B 57: 289-300.

8. Liao G, Wang J, Guo J, Allard J, Cheng J, et al. (2004) In Silico Genetics: Identification of a Functional Element Regulating H2-Ea Gene Expression. Science 306: 690-695.

9. Boyden ED, Dietrich WF (2006) Nalp1b controls mouse macrophage susceptibility to anthrax lethal toxin. Nat Genet 38: 240-244.

10. Yokoyama T, Silversides DW, Waymire KG, Kwon BS, Takeuchi T, et al. (1990) Conserved cysteine to serine mutation in tyrosinase is responsible for the classical albino mutation in laboratory mice. Nucleic Acids Res 18: 7293-7298.

11. Peltz G, Zaas AK, Zheng M, Solis NV, Zhang MX, et al. (2011) Next-Generation Computational Genetic Analysis: Multiple Complement Alleles Control Survival After Candida Albicans Infection Infection and Immunity 79: 4472-4479.

12. Tewhey R, Bansal V, Torkamani A, Topol EJ, Schork NJ (2011) The importance of phase information for human genomics. Nat Rev Genet 12: 215-223.

13. Yang H, Wang JR, Didion JP, Buus RJ, Bell TA, et al. (2011) Subspecific origin and haplotype diversity in the laboratory mouse. Nat Genet 43: 648-655.
